# p300 arrests intervertebral disc degeneration by regulating the FOXO3/Sirt1/Wnt/β‐catenin axis

**DOI:** 10.1111/acel.13677

**Published:** 2022-07-30

**Authors:** Yingjie Hao, Zhinan Ren, Lei Yu, Guangduo Zhu, Panke Zhang, Jian Zhu, Shuyan Cao

**Affiliations:** ^1^ Department of Orthopedics The First Affiliated Hospital of Zhengzhou University Zhengzhou China

**Keywords:** Autophagy, FOXO3, Intervertebral disc degeneration, Nucleus pulposus cells, p300, Sirt1, Wnt/β‐catenin pathway

## Abstract

The transcription factor p300 is reportedly involved in age‐associated human diseases, including intervertebral disc degeneration (IDD). In this study, we investigate the potential role and pathophysiological mechanism of p300 in IDD. Clinical tissue samples were collected from patients with lumbar disc herniation (LDH), in which the expression of p300, forkhead box O3 (FOXO3), and sirtuin 1 (Sirt1) was determined. Nucleus pulposus cells (NPCs) isolated from clinical degenerative intervertebral disc (IVD) tissues were introduced with oe‐p300, oe‐FOXO3, Wnt/β‐catenin agonist 1, C646 (p300/CBP inhibitor), or si‐p300 to explore the functional role of p300 in IDD and to characterize the relationship between p300 and the FOXO3/Sirt1/Wnt/β‐catenin pathway. Also, we established a rat IDD model by inducing needle puncture injuries in the caudal IVDs for further verification of p300 functional role. We found that p300 was downregulated in the clinical tissues and NPCs of IDD. Overexpression of p300 promoted the proliferation and autophagy of NPCs while inhibiting cell apoptosis, which was associated with FOXO3 upregulation. p300 could increase the expression of FOXO3 by binding to the Sirt1 promoter, and thus, contributed to inactivation of the Wnt/β‐catenin pathway. In vivo results further displayed that p300 slowed down the progression of IDD by disrupting the Wnt/β‐catenin pathway through the FOXO3/Sirt1 axis. Taken together, we suggest that p300 can act to suppress IDD via a FOXO3‐dependent mechanism, highlighting a potential novel target for treatment of IDD.

## Graphical abstract

p300 upregulates FOXO3, which combines with the SIRT1 promoter to upregulate SIRT1, leading to inactivation of the Wnt/ß‐catenin pathway, thereby promoting autophagy and inhibiting apoptosis of NPCs to protect against IVDD.

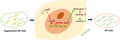

## 1 INTRODUCTION

Intervertebral disc degeneration (IDD) is a disorder of the structural integrity of the discs that interpose between adjoining vertebrae (Fernandez‐Moure et al., 2018). The incidence of IDD is increasing in societies with aging populations (Qian et al., 2019). Indeed, advanced age is the greatest known risk factor for IDD as it is associated with cellular senescence manifesting in reduced proliferative capacities, compromised self‐repair, increased inflammatory responses, and enhanced catabolic metabolism (Vo et al., 2016; Wang et al., 2016). IDD can lead to back, neck, and radicular pain (Risbud & Shapiro, 2014), which greatly diminishes life quality of patients while causing an economic burden to society (Zhang et al., 2018). A decline in the population of nucleus pulposus cells (NPCs) inhabiting the disc is an important characteristic of IDD (Bai et al., 2020). Of note, autophagy of NPCs plays a crucial protective role against IDD and in its treatment (Chen et al., 2016; Chen et al., 2018).

Nuclear phosphoprotein p300, a member of the family of co‐activators including the CREB binding protein CBP, can regulate gene transcription through interaction with components of the transcriptional mechanism (Puri et al., 1997). Interestingly, there is a report in NPCs of transactivation of p300 modulated by PI3K signaling, which can regulate aggrecan gene expression (Cheng et al., 2009). Forkhead box O3 (FOXO3) is a transcription factor with the ability to mediate a variety of physiological processes such as cell behaviors, oxidative stress‐response, as well as energy metabolism (Lutzner et al., 2012). Notably, as previously reported, downregulation of p300 was found to diminish the expression of FOXO3 in murine myotubes cultured in vitro (Kuo et al., 2016). Moreover, FOXO3a can interact with p300 in erythroid progenitor cells (Mahmud et al., 2002). Intriguingly, there is a report of downregulated FOXO3 human degenerated IVDs (Alvarez‐Garcia et al., 2017). In addition, FOXO3 could contribute to the spontaneous development of IDD caused by senescence and loss of cells in the NP (Alvarez‐Garcia et al., 2018). Sirtuin 1 (Sirt1) is the mammalian homolog of yeast Sir2 that belongs to the family of 7 protein and histone deacetylases, which are implicated in multiple biological functions (Wang et al., 2014). Earlier work shows that FOXO3 can induce the expression of Sirt1 in human umbilical endothelial cells (Cho et al., 2013). Sirt1 plays an important role in IDD through its regulation of oxidative stress, inflammatory responses, and mitochondrial function (Zhang et al., 2020). Inhibition of Sirt1 can augment injury of NPCs in an IDD model (Xiang et al., 2020). Of note, Sirt1 activity leads to inactivation of the Wnt/β‐catenin pathway in liver cancer cells (Wu et al., 2017). Previous work shows that the Wnt/β‐catenin pathway can enhance the senescence and apoptosis of NPCs (Zhan et al., 2019). Taken the above findings into consideration, we conducted the current study to in cells and living mice to explore the interaction of p300, FOXO3, and Sirt1 in the development of IDD, and to establish their involvement of the Wnt/β‐catenin pathway.

## 2 RESULTS

### 2.1 Overexpression of p300 alleviated IDD

**FIGURE 1:**
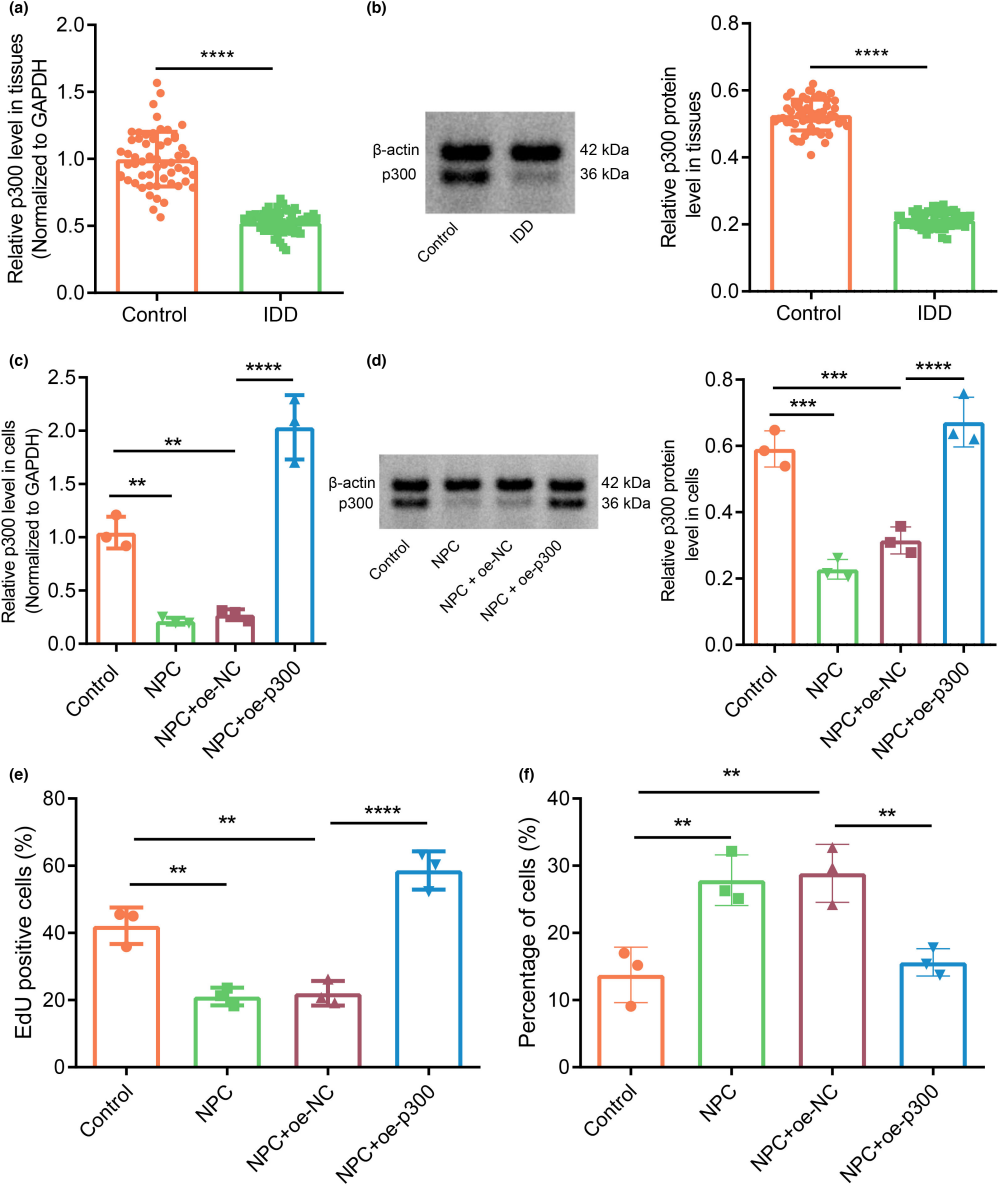
Overexpression of p300 alleviates IDD. (a) p300 expression in clinical NP tissues of healthy controls and patients with IDD as determined by RT‐qPCR (n = 58). *** *p* < 0.001 vs. healthy controls. (b) p300 protein expression in clinical NP tissues of healthy controls and patients with IDD as determined by Western blot assay. *** *p* < 0.001 vs. healthy controls. (c) p300 expression in human NPCs as determined by RT‐qPCR. *** *p* < 0.01 vs. controls. **** *p* < 0.001 vs. NPC + oe‐NC. (d) The protein expression of p300 in NPCs as determined by Western blot assay. *** *p* < 0.001 vs. controls. **** *p* < 0.0001 vs. NPC + oe‐NC. (e) The number of proliferating cells in cultured NPCs as observed by EdU assay. ** *p* < 0.01 vs. controls. **** *p* < 0.0001 vs. NPC + oe‐NC. (f) The proportion of apoptotic cells in cultured NPCs. ** *p* < 0.01 vs. controls

It has been reported that inhibition of p300 aggravates IDD, but the mechanism of p300 participating in IDD needs to be studied (Dong et al., 2019). To further explore the relationship between p300 and IDD, this study enrolled 58 patients (31 males and 27 females; mean age: 52.26 ± 7.19 years) undergone LDH surgery in our hospital. In addition, NP tissues collected from 58 patients (34 males and 24 females; mean age: 51.41 ± 7.30 years) undergoing surgery for thoracolumbar fractures or scoliosis during the same period were selected as a control group. We focused on the L4/L5 or L5‐S1 segments in this project. All NP specimens were stored in liquid nitrogen within 30 min. We graded the control and IDD specimens by the Pfirrmann scale before operation (Table S1). We initially explored the relationship between p300 and IDD in clinical specimens. Reverse transcription‐quantitative polymerase chain reaction (RT‐qPCR) and Western blot assay data showed that p300 expression in the IDD samples was notably lower than that in the control samples (Figure 1a,b). Moreover, RT‐qPCR and Western blot assay data showed that p300 expression in untreated NPCs and NPCs transduced with overexpression‐negative control (oe‐NC) was notably lower than that in controls but was notably higher in NPCs treated with oe‐p300 (Figure 1c,d). EdU assay data showed that cell proliferation of untreated NPCs and oe‐NC‐treated NPCs was markedly lower than that of controls. However, there was an augmentation of proliferation in NPCs in response to oe‐p300 (Figure 1e). In addition, the apoptosis rate of untreated NPCs and oe‐NC‐treated NPCs was enhanced than that of controls, and NPCs treated with oe‐p300 had a lowered apoptosis rate (Figure 1f, Figure S1a). In summary, p300 is downregulated in IDD, whereas its overexpression promotes the proliferation of NPCs and inhibits their apoptosis, thus playing a protective role against IDD.

### 2.2 p300 promoted FOXO3 to inhibit apoptosis of NPCs

**FIGURE 2:**
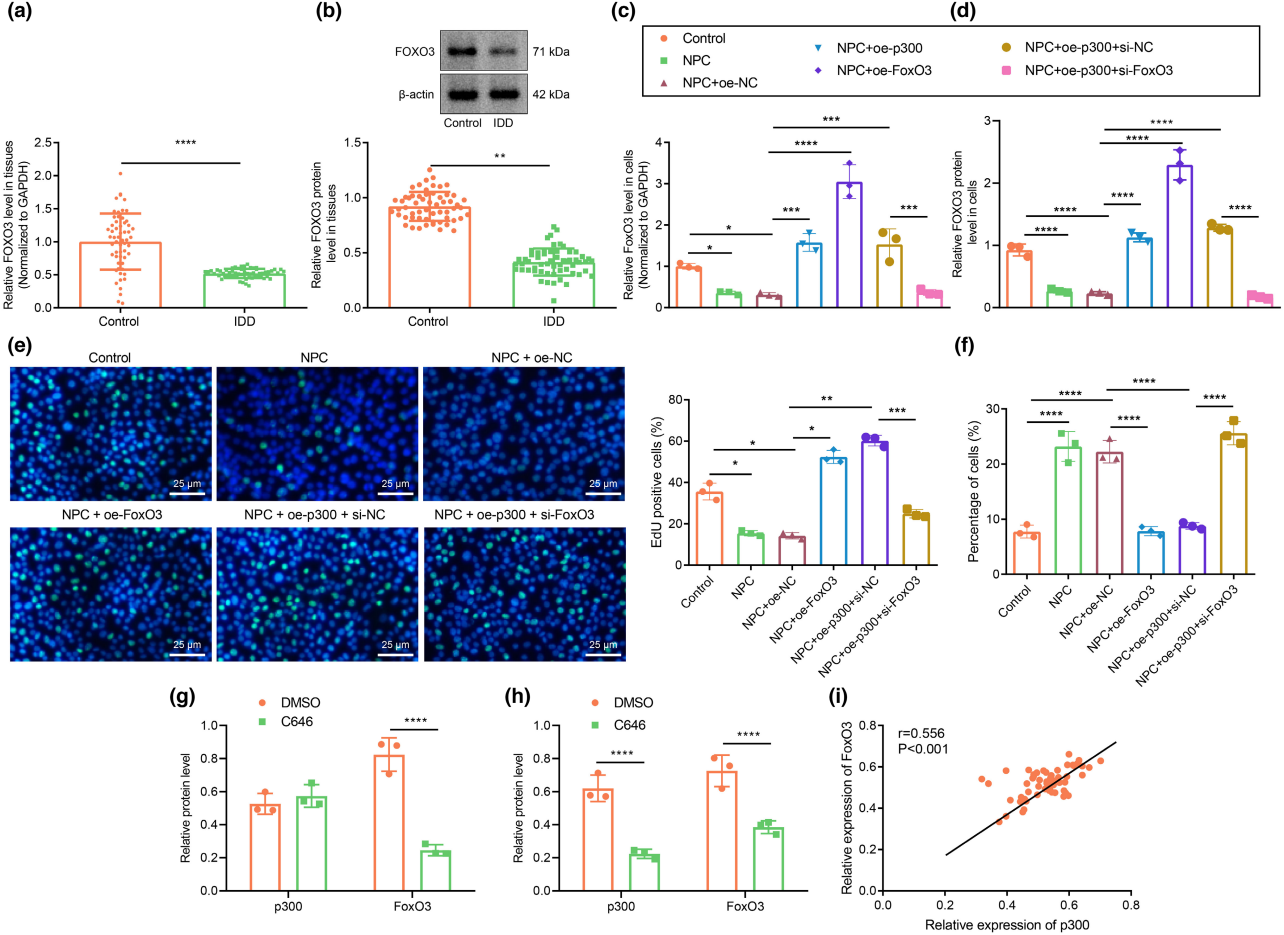
p300 promotes FOXO3 to inhibit apoptosis of NPCs. (a) FOXO3 expression in clinical NP tissues of healthy controls and patients with IDD as determined by RT‐qPCR. n = 58. *** *p* < 0.001 vs. healthy controls. (b) FOXO3 protein expression in clinical NP tissues of healthy controls and patients with IDD as determined by Western blot assay. ** *p* < 0.01 vs. healthy controls. (c) FOXO3 expression in human NPCs as determined by RT‐qPCR. (d) The protein expression of FOXO3 in NPCs as determined by Western blot assay. (e) The number of proliferating cells in cultured NPCs as observed by EdU assay, green fluorescence represents EdU positive staining and blue fluorescence represents DAPI (scale bar: 25 μm). (f) The proportion of apoptotic cells in cultured NPCs. (g) FOXO3 expression in NPCs after inhibition of p300 by C646. *** *p* < 0.001 vs. DMSO. (h) The protein expression of FOXO3 and p300 in NPCs treated with si‐p300 as determined by Western blot assay. (i) Pearson's correlation analysis on the correlation between the expression of p300 and FOXO3 in IVD tissues of IDD patients. The measurement data were expressed as mean ± standard deviation. * *p* < 0.05. ** *p* < 0.01. *** *p* < 0.001. **** *p* < 0.0001. Comparison between two groups was conducted using independent sample *t*‐test. Comparison among multiple groups was conducted using one‐way ANOVA, followed by Tukey post‐hoc test

RT‐qPCR and Western blot assays indicated that FOXO3 expression was appreciably lower in the IDD samples than in the control samples (Figure 2a,b). Furthermore, FOXO3 expression was lower in the clinical tissue samples from IDD patients than in the control samples. Consistently, FOXO3 expression was notably lower in untreated NPCs and NPCs treated with oe‐NC than in control cells but was markedly higher in NPCs in the presence of oe‐p300, oe‐FOXO3 or oe‐p300 + small interfering RNA (si)‐NC. In addition, we noted a decline in the FOXO3 expression in NPCs treated with oe‐p300 + si‐FOXO3 compared with that in NPCs treated with oe‐p300 + si‐NC (Figure 2c,d, Figure S2a). EdU assay data revealed that the proliferation of untreated NPCs and oe‐NC‐treated NPCs was notably lower than that of controls. NPCs treated with oe‐FOXO3, oe‐p300, or oe‐p300 + si‐NC had significantly increased proliferation, whereas NPCs treated with oe‐p300 + si‐FOXO3 had markedly lowered proliferation rate relative to NPCs treated with oe‐p300 + si‐NC (Figure 2e). The flow cytometric data showed an enhanced apoptosis rate in untreated NPCs and oe‐NC‐treated NPCs in comparison with that in controls. The apoptosis rate in NPCs treated with oe‐FOXO3, oe‐p300, or oe‐p300 + si‐NC was notably lower than that in oe‐NC‐treated NPCs, but NPCs treated with oe‐p300 + si‐FOXO3 had markedly increased apoptosis rate versus NPCs treated with oe‐p300 alone (Figure 2f, Figure S1b). Additionally, Western blot analysis showed that the expression of FOXO3 in NPCs was diminished after p300 inhibition with C646 as compared with cells treated with dimethyl sulfoxide vehicle (DMSO), while p300 expression remain unchanged (Figure 2g, Figure S2b). Further silencing of p300 in NPCs diminished the expression of FOXO3 and p300 (Figure 2h, Figure S2c). Person's correlation analysis showed a positive correlation between the expression of FOXO3 with p300 levels in the IVD tissues of IDD patients (Figure 2i). In conclusion, the enhanced expression of p300 can effectively promote the expression of FOXO3 to alleviate IDD.

### 2.3 p300 promoted FOXO3 expression and enhanced autophagy of NPCs

**FIGURE 3:**
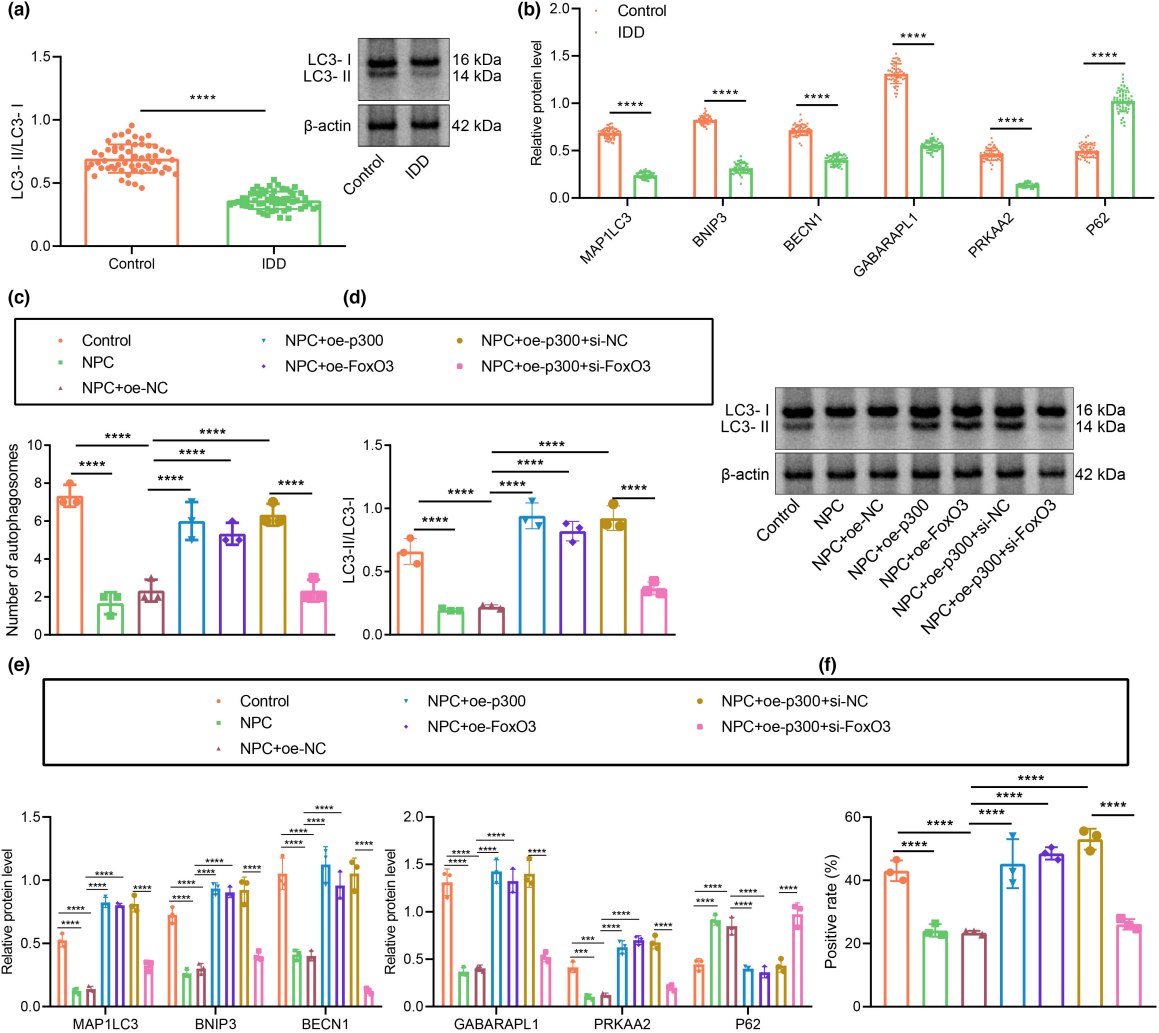
p300 promotes FOXO3 expression and enhances autophagy of NPCs. (a) The ratio of LC3‐II/LC3‐I in clinical samples of healthy controls and patients with IDD as determined by Western blot assay. n = 58. (b) The protein expression of autophagy‐related factors in clinical samples of healthy controls and patients with IDD as determined by Western blot assay. (c) Observation of autophagy in human NPCs by TEM. (d) The ratio of LC3‐II/LC3‐I in NPCs as determined by Western blot assay. (e) The protein expression of autophagy‐related factors in NPCs as determined by Western blot assay. (f) Autophagic flux determined by mRFP‐GFP‐LC3 assay. The expression and location of LC3 in NPCs as examined by immunofluorescence staining. The measurement data were expressed as mean ± standard deviation. * *p* < 0.05. ** *p* < 0.01. *** *p* < 0.001. **** *p* < 0.0001. Comparison between two groups was conducted using independent sample *t*‐test. Comparison among multiple groups was conducted using one‐way ANOVA, followed by Tukey post‐hoc test

Subsequently, we explored whether p300 could affect the autophagy of NPCs by regulating FOXO3 expression. Western blot assay data showed that the ratio of light chain 3 (LC3)‐II to LC3‐I level in degenerative NPCs was notably lower than that in control NPCs (Figure 3a). In addition, we used Western blot assay to measure changes of the autophagy‐related genes. It was found that the protein expression of microtubule‐associated light chain protein 3 (MAP1LC3), Bcl‐2/adenovirus E1B 19‐kDa interacting protein (BNip3), beclin‐1 (BECN1), GABA‐A receptor‐associated protein‐like 1 (GABARAPL1), and AMP‐activated protein kinase alpha 2 (PRKAA2) was markedly lower in the degenerative NPCs, while that of autophagy degradation substrate p62 was higher than that in the control NPCs (Figure 3b, Figure S2d). As observed by transmission electron microscopy (TEM), the number of autophagosomes in the control cells and in NPCs treated with oe‐p300 or oe‐FOXO3 was markedly higher than that in oe‐NC‐treated NPCs (Figure 3c). The ratio of LC3‐II/LC3‐Iand the expression of MAP1LC3, BNip3, BECN1, GABARAPL1, and PRKAA2 in untreated NPCs and oe‐NC‐treated NPCs were notably lower, and that of p62 was higher than that in controls, which were opposite in NPCs treated with oe‐p300 or oe‐FOXO3 compared with results in oe‐NC‐treated NPCs. Conversely, there was a marked reduction in the ratio of LC3‐II/LC3‐I and the expression of MAP1LC3, BNip3, BECN1, GABARAPL1, and PRKAA2, but an increase in that of p62 in NPCs treated with oe‐p300 + si‐FOXO3 relative to NPCs treated with si‐FOXO3 (Figure 3d,e, Figure S2e). In the mRFP‐GFP‐LC3 assay, the yellow (mRFP+‐GFP+) spots in the merged image represent autophagosomes, whereas the red (mRFP+‐GFP) spots indicate the formation of autolysosomes. The number of yellow and red spots was notably lower in untreated NPCs and oe‐NC‐treated NPCs than in controls. oe‐p300 or oe‐FOXO3 treatments significantly increased the numbers of yellow and red spots in the merged image compared with oe‐NC. The numbers of yellow and red spots were diminished in response to oe‐p300 + si‐FOXO3 treatment relative to findings in si‐FOXO3 (Figure 3f). These results suggest that p300 may promote autophagy of NPCs by regulating FOXO3.

### 2.4 FOXO3 bound to the Sirt1 promoter to promote Sirt1 expression in IDD

**FIGURE 4:**
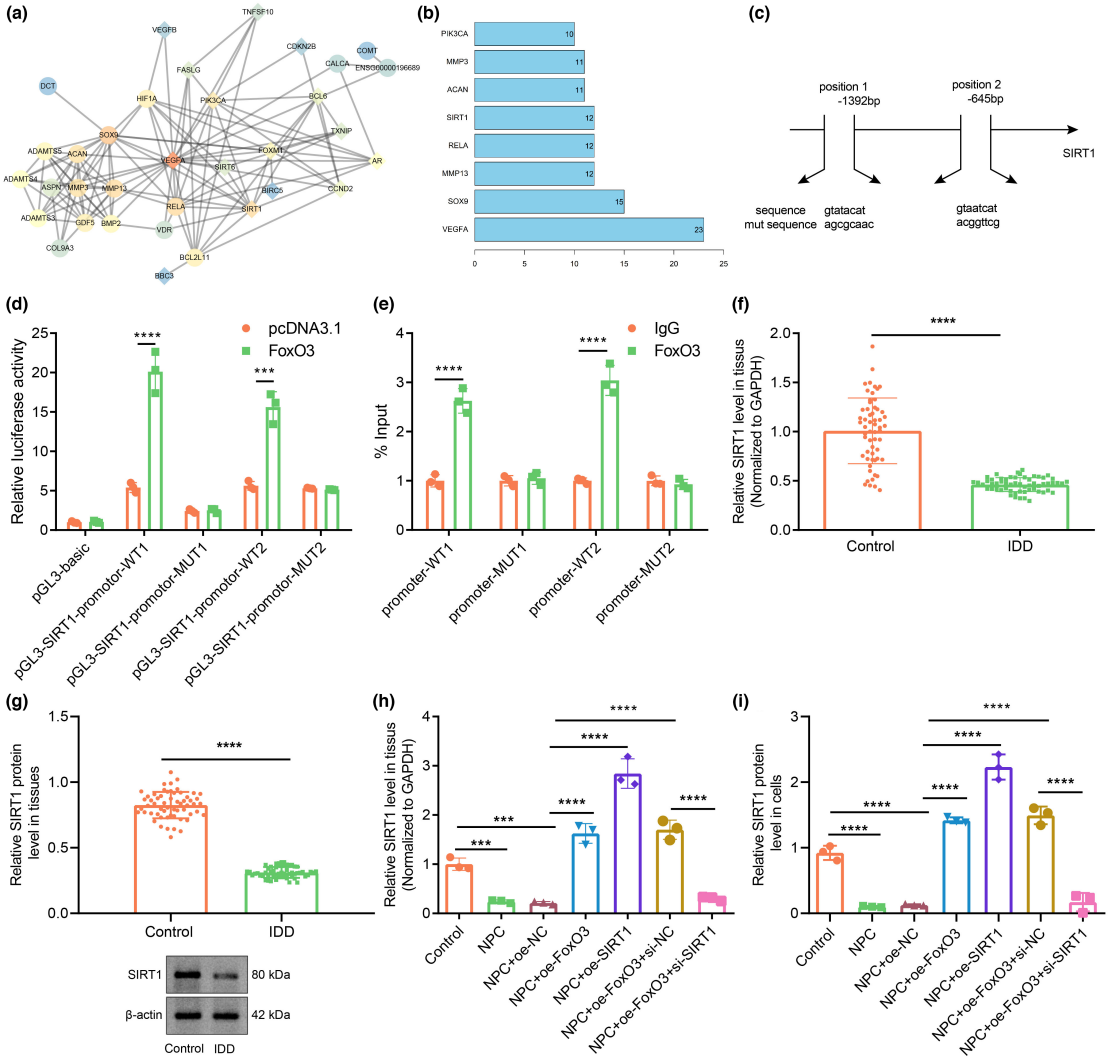
FOXO3 binds to Sirt1 promoter to promote Sirt1 expression in IDD. (a) The interaction network for FOXO3 target genes. (b) The statistical plot for the core value of the core genes. A gene with more interaction genes has a higher core degree value. The X‐axis represents the degree value, and the Y‐axis represents the gene name. (c) The FOXO3 binding site in the Sirt1 promoter and its mutant sequences. (d) The binding relationship between FOXO3 and Sirt1‐promoter as examined by dual‐luciferase reporter gene assay. *** *p* < 0.001 vs. pGL3 SIRT1‐promoter‐WT. **** *p* < 0.0001 vs. pGL3 SIRT1‐promoter‐WT. (e) ChIP‐PCR for FOXO3 enrichment in the SIRT1 promoter. (f) Sirt1 expression in clinical NP tissues of healthy controls and patients with IDD as determined by RT‐qPCR. n = 58. *** *p* < 0.001 vs. healthy controls. (g) Sirt1 protein expression in clinical NP tissues of healthy controls and patients with IDD as determined by Western blot assay. (h) The expression of Sirt1 in NPCs as determined by RT‐qPCR. (i) Sirt1 protein expression in NPCs as determined by Western blot assay. The measurement data were expressed as mean ± standard deviation. * *p* < 0.05. ** *p* < 0.01. *** *p* < 0.001. **** *p* < 0.0001. Comparison was conducted using one‐way ANOVA, followed by Tukey post‐hoc test

In order to understand better the mechanism of FOXO3 in IDD, we searched for genes with a known relationship to IDD using the MalaCards database, and then conducted an interaction analysis between the predicted target genes and known genes to construct a gene–gene interaction network (Figure 4a). Further, VEGFA and Sox9 genes were among those the core position of the network (Figure 4b). Among the set of core genes, Sirt1 was at the core of the whole network, and also had a correlation with most of the known IDD‐related genes. This suggests that Sirt1 may play an important role in the regulation of a gene network of IDD. Furthermore, in the JASPAR database, FOXO3 was predicted to be located in the Sirt1 promoter region, where a binding site was found (Figure 4c). These results and previous reports concur in suggesting that FOXO3 may affect the development of IDD through transcriptional regulation of Sirt1. Dual‐luciferase reporter gene assays verified the predicted binding site of FOXO3 to the Sirt1 promoter (Figure 4d). The experimental data showed that the binding of FOXO3 to Sirt1 promoter‐wild type (WT) was notably higher than that of pcDNA3.1, whereas there was no significant difference between the FOXO3‐Sirt1 promoter‐mutant type (MUT) binding and pcDNA3.1‐Sirt1 promoter‐MUT binding. Chromatin immunoprecipitation (ChIP)‐PCR results showed that Sirt1 enrichment in FOXO3 in the presence of FOXO3 was notably higher than that in the IgG control sample (Figure 4e). RT‐qPCR (Figure 4f) and Western blot assay (Figure 4g) revealed that Sirt1 expression in the IDD samples was notably lower than that in the controls. Moreover, Sirt1 expression underwent a marked decline in oe‐NC‐treated NPCs versus controls. Sirt1 expression in NPCs treated with oe‐FOXO3 or oe‐Sirt1 was notably higher than that in oe‐NC‐treated NPCs, while opposite results were observed in NPCs treated with oe‐FOXO3 + si‐Sirt1 when compared to NPCs treated with si‐Sirt1 (Figure 4h,i, Figure S2f). Overall, these results show that FOXO3 have the potential to promote Sirt1 expression in IDD.

### 2.5 FOXO3 promoted Sirt1 expression to inhibit apoptosis and promote autophagy of NPCs

**FIGURE 5:**
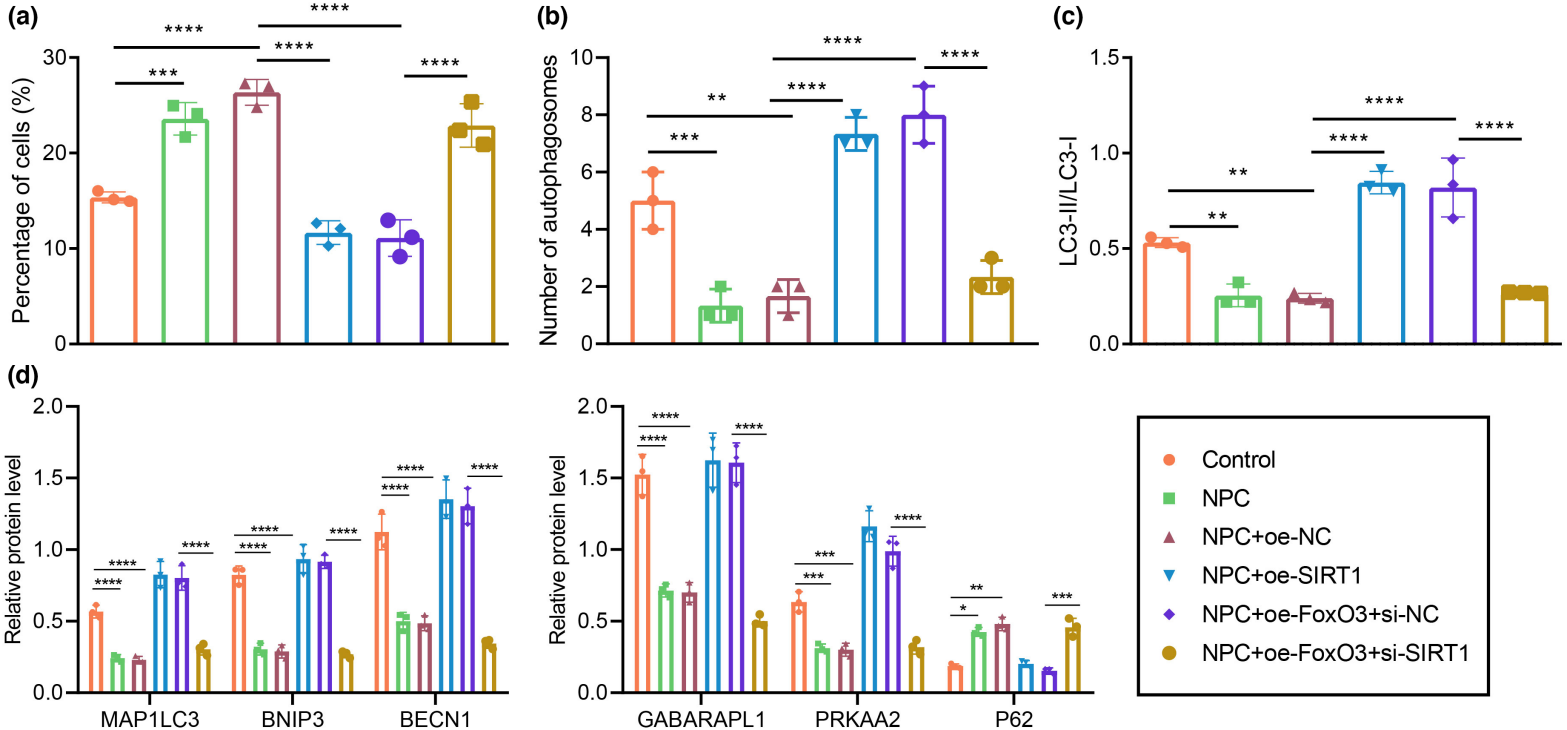
FOXO3 promotes Sirt1 expression to inhibit apoptosis and promote autophagy of NPCs. (a) The proportion of apoptotic cells in cultured NPCs. (b) Observation of autophagosomes in human NPCs by TEM. (c) The changes and ratio of LC3‐II/LC3‐I in NPCs. (d) The expression of autophagy‐related factors in NPCs as determined by Western blot assay. The measurement data were expressed as mean ± standard deviation. * *p* < 0.05. ** *p* < 0.01. *** *p* < 0.001. **** *p* < 0.0001. Statistical comparison was conducted using one‐way ANOVA, followed by Tukey post‐hoc test

Furthermore, we examined the role of FOXO3 in the apoptosis and autophagy of NPCs by regulating Sirt1. Flow cytometry showed that the proportion of apoptotic cells among control untreated NPCs and oe‐NC‐treated NPCs was notably higher than that in controls. Treatments with oe‐FOXO3 + si‐NC or oe‐Sirt1 reversed the increase in proportion of apoptosis of NPCs otherwise seen upon oe‐NC treatment. Compared with oe‐FOXO3 + si‐NC treatments, si‐Sirt1 evoked a notably increased proportion of apoptosis in NPCs (Figure 5a, Figure S1c). As observed by TEM, the number of autophagosomes was elevated in NPCs treated with oe‐Sirt1 or oe‐FOXO3 + si‐NC than that in oe‐NC‐treated NPCs. Relative to oe‐FOXO3 + si‐NC treatment, oe‐FOXO3 + si‐Sirt1 resulted in a decline in the number of autophagosomes in NPCs (Figure 5b).

As shown by Western blot assay, in comparison with oe‐NC‐treated NPCs, the ratio of LC3‐II/LC3‐I and the expression of MAP1LC3, BNip3, BECN1, GABARAPL1, and PRKAA2 were elevated, but that of p62 was lower in NPCs treated with oe‐Sirt1 or oe‐FOXO3 + si‐NC. Relative to oe‐FOXO3 + si‐NC, treatment with oe‐FOXO3 + si‐Sirt1 led to a decline in the ratio of LC3‐II/LC3‐I and reduced expression of MAP1LC3, BNip3, BECN1, GABARAPL1, and PRKAA2, but increase in that of p62 (Figure 5c,d, Figure S2g,h). Taken together, FOXO3 can promote Sirt1 expression to inhibit apoptosis and promote autophagy of NPCs.

### 2.6 p300‐mediated FOXO3 promoted Sirt1 to inhibit apoptosis of NPCs through inactivation of the Wnt/β‐catenin pathway

**FIGURE 6:**
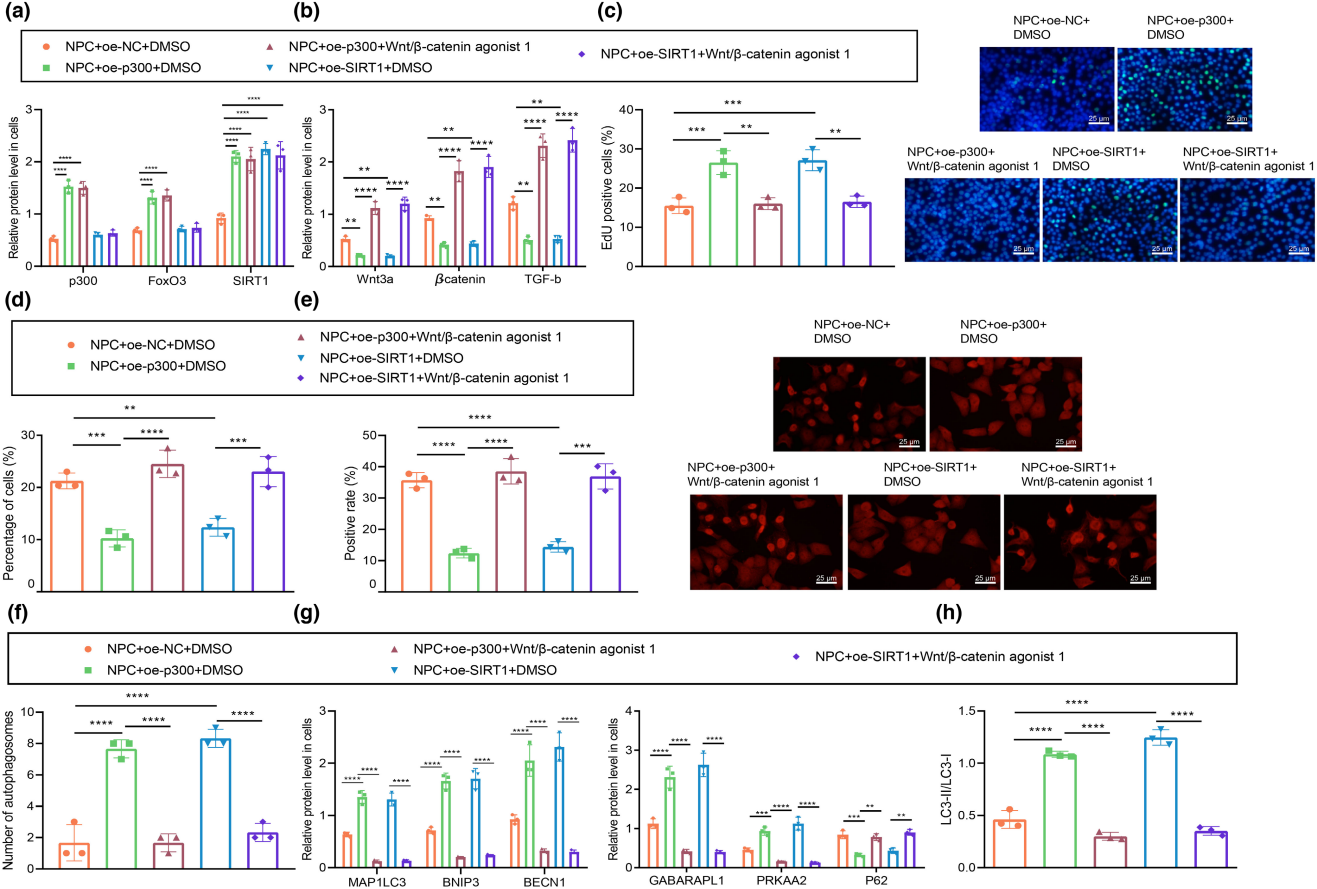
p300‐mediated FOXO3 promotes Sirt1 to inhibit apoptosis of NPCs through inactivation of the Wnt/β‐catenin pathway. (a) Protein expression changes of p300, FOXO3 and Sirt1 in NPCs with different treatment as determined by Western blot assay. (b) The expression of Wnt/β‐catenin pathway‐related factors in NPCs after treatment with Wnt/β‐catenin agonist 1 as determined by Western blot assay. (c) The number of proliferating cells in cultured NPCs as observed by EdU staining (scale bar: 25 μm), green fluorescence represents EdU positive staining and blue fluorescence represents DAPI. (d) The proportion of apoptotic cells in NPCs. * *p* < 0.05 vs. NPCs treated with oe‐NC + DMSO. # *p* < 0.05 vs. NPCs treated with oe‐p300 + DMSO. $ *p* < 0.05 vs. NPCs treated with oe‐Sirt1 + DMSO. (e) Immunofluorescence staining for determination of the nucleation of β‐catenin in NPCs (scale bar: 25 μm), red fluorescence represents β‐catenin positive staining and blue fluorescence represents DAPI. (f) The autophagy in human NPCs as observed by TEM. (g) The protein expression of autophagy‐related factors in NPCs after treatment with Wnt/β‐catenin agonist 1 as determined by Western blot assay. (h) The protein expression of LC3‐II and LC3‐1 and the changes in the ratio of LC3‐II/LC3‐1 in NPCs after treatment with Wnt/β‐catenin agonist 1 as determined by Western blot assay. The measurement data were expressed as mean ± standard deviation. * *p* < 0.05. ** *p* < 0.01. *** *p* < 0.001. **** *p* < 0.0001. Statistical comparison was conducted using one‐way ANOVA, followed by Tukey post‐hoc test

We then examined the role of the FOXO3/Sirt1 axis in IDD, with the involvement of Wnt/β‐catenin. Western blot assay results showed that the protein expression of p300, FOXO3, and Sirt1 was significantly increased in NPCs overexpressing p300, while overexpression of SIRT1 (as expected) only upregulated SIRT1 protein expression (Figure 6a, Figure S3a). Moreover, the protein expression of Wnt/β‐catenin pathway‐related factors in NPCs showed a decline in response to oe‐p300 + DMSO treatment relative to oe‐NC + DMSO treatment. In contrast, the protein expression of Wnt/β‐catenin pathway‐related factors in NPCs upon exposure to Wnt/β‐catenin agonist 1 was notably higher than that in the presence of oe‐p300 + DMSO. NPCs treated with oe‐Sirt1 + Wnt/β‐catenin agonist 1 showed an increase in the protein expression of Wnt/β‐catenin pathway‐related factors compared with that in cells treated with oe‐Sirt1 + DMSO (Figure 6b, Figure S3b). The EdU assay and flow cytometry results showed that the proliferation of NPCs increased while apoptosis declined in response to oe‐p300 + DMSO or oe‐Sirt1 + DMSO treatment relative to results in the oe‐NC + DMSO treatment group. The proliferation of NPCs upon oe‐p300 + Wnt/β‐catenin agonist 1 treatment was lowered, while the apoptosis rate was enhanced than corresponding results in response to oe‐p300 + DMSO treatment. NPCs treated with oe‐p300 + Wnt/β‐catenin agonist 1 or oe‐Sirt1 + Wnt/β‐catenin agonist 1 revealed diminished proliferation and increased apoptosis as comparted to findings in NPCs treated with oe‐Sirt1 + DMSO (Figure 6c,d, Figure S1d). Based on the results from immunofluorescence staining, expression of the Wnt/β‐catenin pathway‐related factors in the nuclei of NPCs displayed a decline in response to oe‐p300 + DMSO or oe‐Sirt1 + DMSO treatments relative to oe‐NC + DMSO. Conversely, the expression of the Wnt/β‐catenin pathway‐related factors in the nuclei of NPCs upon oe‐p300 + Wnt/β‐catenin agonist 1 was notably higher than that in response to oe‐p300 + DMSO. NPCs treated with oe‐Sirt1 + Wnt/β‐catenin agonist 1 revealed elevated expression of the Wnt/β‐catenin pathway‐related factors in the nuclei as compared to cells treated with oe‐Sirt1 + DMSO (Figure 6e). As revealed by TEM, the number of autophagosomes was increased in response to oe‐p300 + DMSO or oe‐Sirt1 + DMSO versus oe‐NC + DMSO, but autophagosome counts were lower upon treatment with oe‐p300 + Wnt/β‐catenin agonist 1 or oe‐Sirt1 + Wnt/β‐catenin agonist 1 (Figure 6f). Furthermore, there was increased protein expression of MAP1LC3, BNip3, BECN1, GABARAPL1, and PRKAA2, but reduced expression of p62 in the presence of oe‐p300 + DMSO or oe‐Sirt1 + DMSO relative to oe‐NC + DMSO. However, these results were opposite in the presence of oe‐p300 + Wnt/β‐catenin agonist 1 or oe‐SIRT1 + Wnt/β‐catenin agonist 1 relative to their separate controls, that is, oe‐p300 + DMSO and oe‐SIRT1 + DMSO (Figure 6g, Figure S3c). The expression of LC3‐II and the ratio of LC3‐II/LC3‐I increased in the presence of oe‐p300 + DMSO or oe‐Sirt1 + DMSO relative to that upon oe‐NC + DMSO treatment. However, these markers were lower upon treatment with oe‐p300 + Wnt/β‐catenin agonist 1 or oe‐SIRT1 + Wnt/β‐catenin agonist 1 relative to their separate controls, that is, oe‐p300 + DMSO and oe‐SIRT1 + DMSO (Figure 6h, Figure S3d). Taken together, these results suggest that overexpression of p300 can promote the expression of FOXO3 and activate Sirt1, thus inhibiting the expression of factors in the Wnt/β‐catenin pathway and then promoting autophagy of NPCs.

### 2.7 Overexpression of p300 regulates the Wnt/β‐catenin pathway to inhibit IDD in rats

**FIGURE 7:**
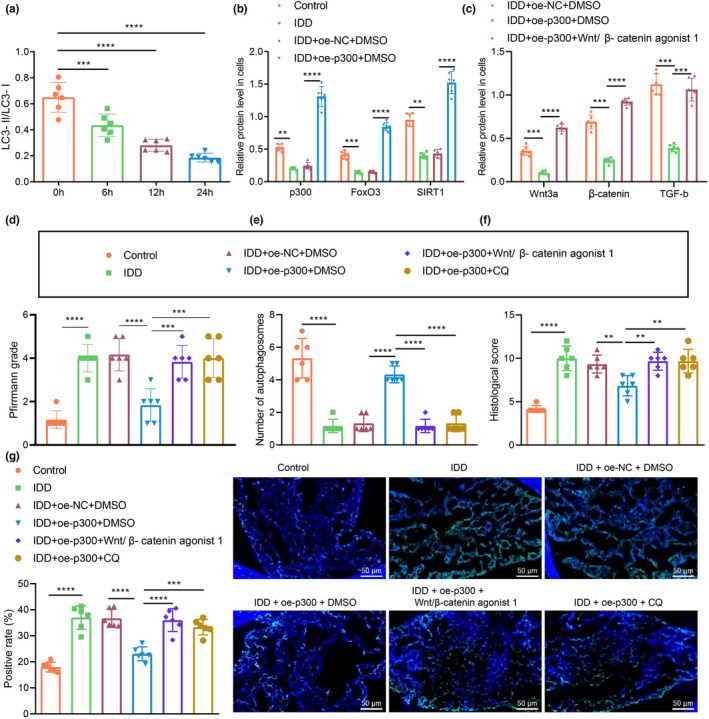
Overexpression of p300 regulates the Wnt/β‐catenin pathway to inhibit IDD in rats. (a) LC3‐II/LC3‐I ratio in the IVD tissues of IDD rats as determined by Western blot assay. (b) Changes of protein expression of p300, FOXO3 and Sirt1 in the IVD tissues of IDD rats as determined by Western blot assay. (c) Changes of protein expression of Wnt/β‐catenin pathway‐related factors in the IVD tissues of IDD rats as determined by Western blot assay. (d) IDD in rats determined by the MRI method with Pfirrmann grading. (e) Autophagosomes in NPCs of rat IVD tissues as observed by TEM. (f) Safranin O‐fast green staining for the pathological changes of IVD tissues. (g) TUNEL staining for the apoptosis of rat IVD tissues (scale bar: 50 μm), green fluorescence represents TUNEL staining and blue fluorescence represents DAPI. The measurement data were expressed as mean ± standard deviation. * *p* < 0.05. ** *p* < 0.01. *** *p* < 0.001. **** *p* < 0.0001. Statistical comparison was conducted using one‐way ANOVA, followed by Tukey post‐hoc test. There were 6 rats in each group

Finally, we aimed to characterize the effect of p300 on IDD in the established Sprague‐Dawley rat IDD model. We used Western blot assay to measure the protein expression of LC3‐II and LC3‐I in IVD tissues of rats in response to autophagy inhibitor CQ (Sigma‐c6628) at different time points. The results revealed that CQ could successfully reduce the LC3‐II/LC3‐I ratio (Figure 7a, Figure S3e), and confirmed the expected reduction of p300, FOXO3, and Sirt1 expression in IVD tissues of IDD rats compared with that in of control rats. Conversely, the protein expression of p300, FOXO3, and Sirt1 in IVD tissues of IDD rats in the presence of oe‐p300 + DMSO was notably higher than that in those in the presence of oe‐NC + DMSO (Figure 7b, Figure S3f). Moreover, IDD rats treated with oe‐p300 alone had notably diminished protein expression of Wnt/β‐catenin pathway‐related factors in IVD tissues. Versus IDD rats treated with oe‐p300 alone, IDD rats in response to oe‐p300 + Wnt/β‐catenin agonist 1 presented augmented protein expression of Wnt/β‐catenin pathway‐related factors (Figure 7c, Figure S3g). In addition, Pfirrmann grading scores were higher in untreated IDD rats or oe‐NC + DMSO‐treated IDD rats than in control rats. The Pfirrmann score was diminished in IDD rats in the presence of oe‐p300 alone, while IDD rats treated with oe‐p300 + Wnt/β‐catenin agonist 1 or oe‐p300 + CQ demonstrated higher Pfirrmann grading in comparison with the oe‐p300‐treated IDD rats (Figure 7d, Figure S4). TEM examination showed a notable increase in the number of autophagosomes in oe‐p300‐treated IDD rats, while the additional treatments of Wnt/β‐catenin agonist 1 or CQ reversed the increase in autophagosome counts (Figure 7e, Figure S5). Histological examination by safranin O‐fast green staining showed markedly lower scores of untreated IDD rats or oe‐NC + DMSO‐treated IDD rats when compared to control rats. Moreover, IDD rats in the presence of oe‐p300 alone had significantly diminished histological scores, while IDD rats upon additional treatments of Wnt/β‐catenin agonist 1 or CQ had markedly increased histological scores (Figure 7f, Figure S6). TUNEL staining results showed an elevated number of apoptotic cells in untreated IDD rats or oe‐NC + DMSO‐treated IDD rats as comparted to control rats. However, IDD rats treated with oe‐p300 alone had fewer apoptotic cells, while additional treatment of Wnt/β‐catenin agonist 1 or CQ treatments resulted in a notably increased apoptosis rate (Figure 7g). In summary, overexpression of p300 can inhibit the Wnt/β‐catenin pathway in vivo and protect against IDD. CQ treatment, an autophagy inhibitor, could counteract that protective effect of p300 treatment.

## 3 DISCUSSION

IDD, a leading contributor to various chronic and debilitating disorders of the spine, and its pathophysiology remains unclarified (Bai et al., 2017). In this study, we attempted to investigate in what measure p300, FOXO3, and Sirt1 might affect the development of IDD via regulation of the Wnt/β‐catenin pathway; our results showed that p300‐mediated FOXO3 increases in Sirt1 inhibited the Wnt/β‐catenin pathway, which promotes autophagy and inhibits apoptosis of NPCs.

In the first phase of the study, we revealed downregulation of p300 expression in tissue sample from patients with IDD and showed that overexpression of p300 inhibited the apoptosis of NPCs. Moreover, we verified that this effect occurred due to upregulation of FOXO3 by p300. In fact, previous work showed that p300 plays a role in autophagy and disease progression. The acetyltransferase activity of p300 determines the acetylation of a variety of components of the autophagy system (Lee & Finkel, 2009). An increased p300 activity due to AKG leads to an increase in autophagy, thereby extending the lifespan of Drosophila (Su et al., 2019). Furthermore, there are previous studies partly in support of our findings that p300 upregulates FOXO3 expression. For instance, Jeung et al. demonstrated that overexpression of p300 could elevate FOXO3a levels in lung cancer cells (Jeung et al., 2016). Furthermore, Wang et al. found that FOXO3a could bind to the KIX domain of CBP/p300 at MLL and to c‐Myb binding sites in a concurrent manner (Wang et al., 2012). Overall, we demonstrated that the upregulation of FOXO3 was a key aspect of the protective role of p300 against IDD.

Another crucial finding obtained in this study is that FOXO3 activated Sirt1 promoter, and thus, stimulated Sirt1 expression. A large number of studies have highlighted the inhibitory role of FOXO3 in the development of IDD. For example, others have shown that the restoration of FOXO3 expression by antagomiR‐221 could ameliorate the severity of IDD (Penolazzi et al., 2018). In addition, the activation by tSIRT3 of the SIRT3/FOXO3/SOD2 pathway by tSIRT3 was capable of retarding the progression of IDD in an animal model (Zhou et al., 2019). Besides, Wang et al. identified the protective role of FOXO3 against the apoptosis of NPCs through autophagy (Wang et al., 2020). Thus, Sirt1 has emerged as an inhibitory factor for IDD. Indeed, the miR‐182‐5p/Sirt1 axis could alleviate IDD through regulation of mitophagy and apoptosis (Xie et al., 2019), whereas conversely, the depletion of Sirt1 due by miR‐141 treatment promoted IDD by inducing the apoptosis of NPCs (Ji et al., 2018). In accordance with our present findings, previous studies have demonstrated the interaction between FOXO3 and Sirt1. Intriguingly, Hubbard et al. discovered that FOXO3a could promote the activation of Sirt1 by sirtuin‐activating compounds (Hubbard et al., 2013). Besides, FOXO3 could upregulate Sirt1 expression in human umbilical endothelial cells (Cho et al., 2013). This background is in general agreement with our present finding that FOXO3 contributed to increased Sirt1 expression, thereby promoting the autophagy of NPCs to protect against IDD.

We further extended our mechanistic study to show that Sirt1 could inhibit the Wnt/β‐catenin pathway in NPCs. Intriguingly, there are increasing reports on the regulatory function of Sirt1 on the Wnt/β‐catenin pathway. For instance, FOXO transcription factors could inactivate the Wnt/β‐catenin signaling in osteoblast progenitors, thereby promoting cortical bone formation (Iyer et al., 2014). Moreover, Sirt1 could downregulate the Wnt/β‐catenin pathway to inhibit microglial activation in the aftermath of spinal cord injury (Lu et al., 2019). Furthermore, accumulating evidence shows that the Wnt/β‐catenin pathway participates in the pathogenesis of IDD. In line with our findings, a previous study showed that inactivation of the Wnt/β‐catenin pathway by microRNA‐185 treatment could contribute to an alleviation of IDD (Yun et al., 2020). Additionally, the canonical Wnt/β‐catenin pathway participated in the development of IDD in part by accelerating the senescence of NPCs (Liu et al., 2020).

Taken together, the current study results demonstrate that p300 could elevate the expression of FOXO3, which then bound to the SIRT1 promoter, and thus, increasing SIRT1 expression, thereby inhibiting the Wnt/β‐catenin pathway. By this mechanism, there was a promotion of NPC autophagy and attenuation of cell apoptosis, accompanied by suppression of IDD progression. These results suggest that p300 has a potent protective effect against IDD, which may present a possible therapeutic channel for the treatment of IDD and other age‐related degenerative diseases. This prospect calls for further research aiming to establish the clinical feasibility of such an approach.

## 4 MATERIALS AND METHODS

### 4.1 ETHICS STATEMENT

The study was performed with the approval of the Ethics Committee of the First Affiliated Hospital of Zhengzhou University and performed in strict accordance with the *Declaration of Helsinki*. All participants signed informed consent documentation before sample collection. Animal experiments were approved by the Animal Ethics Committee of the First Affiliated Hospital of Zhengzhou University and conducted strictly following the Guide for the Care and Use of Laboratory Animals published by the US National Institutes of Health. Extensive efforts were made to ensure minimal suffering of the included animals.

### 4.2 Bioinformatics analysis

Through the TRRUST version 2 database, we predicted the potential downstream target genes of FOXO3. The MalaCards database was used to retrieve the known genes related to IDD. The STRING database was utilized to analyze interactions between target genes and the known genes, and to construct a gene–gene interaction network. Binding sites between FOXO3 and the Sirt1 promoter region were predicted using the JASPR database.

### 4.3 Clinical sample collection

This study enrolled 58 patients (31 males and 27 females; mean age: 52.26 ± 7.19 years) who had undergone lumbar disc herniation (LDH) surgery in the First Affiliated Hospital of Zhengzhou University. In addition, NP tissues collected from 58 patients (34 males and 24 females; mean age: 51.41 ± 7.30 years) undergoing surgery for thoracolumbar fractures or scoliosis during the same period were selected as a control group. We focused on the L4/L5 or L5‐S1 segments in this project. All NP specimens were stored in liquid nitrogen within 30 min. We graded the control and IDD specimens by the Pfirrmann scale before operation (Table S1). Specific clinical information is shown in Table S2. Each specimen was divided into two parts; one part was immediately used obtain primary NPCs, and the other sample was stored at −80°C refrigerator for later protein and RNA extraction.

### 4.4 Isolation and culture of human NPCs

NP tissue samples were isolated and sliced, and treated with 0.25% pronase (Sigma) for 30 min and 0.2% collagenase II (Invitrogen) for 4 h at 37°C. Digestion was carried out on a 70 μm well size grid and the cells were then cultured in 5% carbon dioxide at 37°C with Dulbecco's modified Eagle's medium (DMEM) (Gibco) containing 10% fetal bovine serum (FBS), 1% penicillin–streptomycin, 2 mM glutamine, and 50 μg/ml L‐ascorbic acid. When the cells grew to confluence, they were detached with 0.25% trypsin containing 1 mM EDTA. Cells at passage 2 were collected and used for all experiments. Using the primary cells as controls, passage 2 cells were collected for immunohistochemical staining for collagen type II. The increased expression of type II collagen and Sox9 as determined by RT‐qPCR served for identification of NPCs. After cell transfection with 30–50% lentivirus (Invitrogen) for 12 h, 95% of the cells survived. The transfection effect was examined by Western blot assay. The cells were assigned into controls (normal NPCs as controls), NPCs (NPCs from patients with IDD), or NPCs transfected with oe‐NC (NPCs overexpressing NC), oe‐p300 (NPCs overexpressing p300), oe‐FOXO3 (NPCs overexpressing FOXO3), oe‐NC + DMSO (NPCs overexpressing NC and treated with DMSO), oe‐p300 + DMSO (NPCs overexpressing p300 and treated with DMSO), oe‐p300 + Wnt/β‐catenin agonist 1 (NPCs overexpressing p300 and treated with Wnt/β‐catenin agonist 1), C646 (NPCs treated with a p300/CBP inhibitor C646 [ab142163, Abcam Inc.]), and si‐p300 (NPCs treated with si‐p300).

### 4.5 RT‐qPCR

Total RNA was extracted from cells using TRIzol reagent (16,096,020, Thermo Fisher Scientific), 5 μg of which was synthesized into complementary DNA (cDNA) through reverse transcription according to the instructions of the cDNA kit (K1622; Fermentas Inc.). Using cDNA as the template, RT‐qPCR was performed following the TaqMan Gene Expression Assays Protocol (Applied Biosystems). Glyceraldehyde‐3‐phosphate dehydrogenase (GAPDH) served as the internal reference for mRNA. Three duplicated wells were set for each RT‐qPCR assay. The primer design is depicted in Table S3. We used the 2^−ΔΔCT^ method to compare between the experimental and the control groups.

### 4.6 Western blot assay

Total protein was extracted with radio‐immunoprecipitation assay buffer (RIPA) kits (R0010; Solarbio), and the protein concentration was determined using a bicinchoninic acid (BCA) protein assay kit (GBCBIO Technologies). A total of 40 μg protein from each sample was separated by gel electrophoresis with 10% sodium dodecyl sulfate‐polyacrylamide gel. The protein was then transferred to a polyvinylidene fluoride membrane (Millipore), which was sealed with Tris‐buffered saline‐Tween 20 (TBST) solution containing 5% bovine serum albumin (BSA) at room temperature. Next, the protein was incubated with diluted primary antibodies to p300 (1: 1000, ab54984), FOXO3a (1: 1000, ab70314), Sirt1 (1: 1000, ab110304), microtubule‐associated protein LC3 (1: 1000, ab128025), Wnt3a (1: 1000, ab28472), β‐catenin (1: 1000, ab6302), transforming growth factor β1 (TGF‐β1) (1: 500, ab92486), MAP1LC3 (1: 2000, ab52628), BNip3 (1: 5000, ab10433), BECN1 (1: 5000, ab231341), GABARAPL1 (1: 1000, ab86497), PRKAA2 (1: 1000, ab3760, 63 kDa), and p62 (1: 1000, ab56416) overnight at 4°C. The following day, the secondary antibody goat anti‐rabbit against immunoglobulin G (IgG) (ab97051, 1: 2000) or goat anti‐mouse against IgG (ab205719, 1: 2000) was added to the protein for incubation at room temperature. The protein bands were developed by enhanced chemiluminescence, followed by imaging on an Image Quant LAS 4000C gel imager (GE company).

### 4.7 EdU assay

The EdU assay was applied to directly measure DNA synthesis for cell proliferation determination. NPCs were isolated from the co‐culture system, washed twice with phosphate‐buffered saline (PBS), and seeded into 96‐well plate at a density of 5 × 10^3^ cells/well. After 6 h, the cells were labeled with EdU and incubated in EdU medium for 2 h (100 μl/well). Fixative (4% paraformaldehyde) was added to the plate (100 μl/well), followed by incubation for 30 min at room temperature. Next, 2 mg/ml glycine was added to the cells for 5 min incubation (100 μl/well), followed by addition of 100 μl/well penetrant (0.5% Triton X‐100 PBS) with incubation for 10 min. Subsequently, the cells were stained with 100 μl/well 1 × Apollo staining reaction solution in the dark for 30 min. Afterwards, 1 × Hoechst 33342 reaction solution was added to the plate (100 μl/well), followed by incubation for 30 min in a decolorizing shaker at room temperature. After staining, an anti‐fade agent was added to the plate (100 μl/well) and the number of cells labeled by EdU was recorded. Here, the positive and negative cells were counted in three randomly selected fields under microscopy, and the EdU labeling rate (%) was calculated as the number of positive cells / (the number of positive cells + the number of negative cells) × 100%.

### 4.8 Flow cytometry

Flow cytometry was used to measure cell apoptosis. In this experimental procedure, at 48 h after transfection, the medium of each of cell culture plate was transferred into a 15 ml conical tube and placed on ice. The cells remaining in the culture plate were moistened with 2 ml PBS solution; 0.5 ml of 0.25% trypsin was added to the cells, which were incubated until they began to fall off from the culture plate wall under microscopic examination. The cells were completely detached from the culture plate wall by gentle and continuous trituration. The cells were gently resuspended in the previously collected medium to a density of about 1 × 10^6^ cells/ml Portions of 0.5 ml cell suspension (5 × 10^5^ cells) were transferred from the cell culture plate to a clean centrifuge tube, followed by addition of staining solution. The cells were gently resuspended with 0.5 ml of precooled 1 × binding buffer, and incubated with 5 μl Annexin V‐fluorescein isothiocyanate conjugate (FITC) and 10 μl propidium iodine (PI) in the dark for 15 min. A flow cytometer (BD Biosciences) was used for cell measurement and analysis. All the above reagents were from Beyotime.

### 4.9 TEM

TEM was used for autophagosome observation. NPCs were fixed overnight in 2.5% glutaraldehyde, and then in 2% osmium tetroxide for 1 h and stained with 2% uranyl acetate for another 1 h. The NPCs were dehydrated in an acetone concentration gradient, embedded in epoxy resin, and cut into semi‐thin sections, which were first located using toluidine blue staining. The cells were observed in 80 nm ultra‐thin sections by TEM (Hitachi).

NPCs (1 mm^3^) from L6 to L7 were obtained. The samples were placed in 2% glutaraldehyde at room temperature and treated with 0.1 M sodium cacodylate buffer (pH 7.4) for 2 days. Following three washes with 0.1 M PBS, the samples were treated with 0.1 M sodium cacodylate buffer for repair, and then immersed in 1% OsO4 (pH 7.4) at 4°C for 12 h. The samples were dehydrated by gradient ethanol and embedded in epoxy resin. Finally, the embedded samples were sectioned (60 nm) and stained with 2% uranyl acetate for 1 h. The ultra‐thin sections were observed by a TEM.

### 4.10 Immunofluorescence staining

Immunofluorescence staining was employed to analyze the localization and expression of related proteins in NPCs. Cells were placed into a 35‐mm cell culture dish, fixed with 4% cold paraformaldehyde for 20 min, and permeated with 0.2% Triton X‐100 for 10 min. The cells were then blocked with serum of the same host as the secondary antibody for 30 min. The primary antibody (β‐catenin: 1: 300, ab32572, Abcam) was used to incubate the cells in a wet box at 4°C overnight. The secondary antibodies (ab150115) were added to the cells for 2‐h incubation at room temperature in darkness. Finally, DAPI was used to dye the nucleus, and then the fluorescent films were directly photographed, followed by blockade with glycerin and observation under a fluorescence microscope.

### 4.11 Tandem fluorescent‐tagged LC3 (mRFP‐GFP‐LC3) assay for monitoring autophagic flux

The mRFP‐GFP‐LC3 plasmid was used to establish a dual‐fluorescence autophagy system to evaluate the autophagy flux. The lentivirus containing the HBLV‐mRFP‐GFP‐LC3‐PURO plasmid was purchased from Hansheng Biotechnology (Shanghai, China). NPCs were transduced with lentivirus to induce stable expression of mRFP‐GFP‐LC3 and screened with 1 μg/mL puromycin. A fluorescence microscope (Olympus BX51, Tokyo, Japan) was used to detect red (RFP) and green (GFP) fluorescence. Since LC3 accumulated in autophagosomes, they showed red (mRFP) and green (GFP) fluorescence simultaneously. The yellow (mRFP+‐GFP+) spots in the merged image represented autophagosomes. However, in autophagy flux conditions, lysosomes fused with autophagosomes, and the green (mGFP) fluorescence was quenched by the acidic microenvironment. Therefore, the red (mRFP+‐GFP) spots in the merged image indicated the formation of autolysosomes. The autophagy flux of NPCs was observed using a fluorescence microscope.

### 4.12 Dual‐luciferase reporter gene assay

Dual‐luciferase reporter gene assay was used to verify the binding of FOXO3 to the SIRT1 promoter. The Sirt1 promoter region was cloned into psiCHECK‐2 (Promega Corporation, Madison, WI) luciferase vector to construct the Sirt1‐promoter‐WT (Sirt1 promoter plasmid of WT), Sirt1‐promoter‐MUT1 (Sirt1 promoter plasmid of MUT1), Sirt1‐promoter MUT2 (Sirt1 promoter plasmid of MUT2). HEK‐293 T cells were seeded into a 24‐well plate. After 24 h in culture, 100 ng of FOXO3 control plasmid (oe‐NC) or 100 ng of FOXO3‐encoding plasmid (oe‐FOXO3) were mixed with 50 nM of Sirt1 promoter plasmid based on the protocol in the Lipofectamine kit 2000 (Invitrogen). Luciferase activity was measured 48 h after transfection. PCR primers for the Sirt1 promoter are shown in Table S4.

### 4.13 ChIP‐PCR

ChIP‐PCR was applied for determination of FOXO3 binding to the SIRT1 promoter. After the cells were treated with 4% formaldehyde to a final concentration of 1%, the collected cells were ultrasonically disrupted. Rabbit anti‐human against FOXO3 (1: 1000, ab70314, Abcam) was added to the suspension to combine with the FOXO3‐Sirt1 promoter complex. Next, Protein A Agarose/SaLmon Sperm DNA was added to the cells to combine with the FOXO3 antibody‐FOXO3‐Sirt1 promoter complex. The precipitated complex was washed to remove some of the nonspecific binding. After elution, the enriched FOXO3‐Sirt1 promoter complex was obtained, which was subjected to de‐crosslinking. The enriched Sirt1 promoter fragments were purified and analyzed by PCR. The sequences of PCR primers are shown in Table S4.

### 4.14 Establishment of the IDD rat model and animal treatment

IDD was simulated via needle puncture injuries in the caudal IVDs in vivo as described previously (Li et al., 2020; Yang et al., 2019). In this experiment, we assigned 3‐month‐old male Sprague Dawley rats into 8 groups, which were operated to establish the IDD models with various treatments or were un‐operated to serve as NC (Cheng et al., 2018). After induction of anesthesia, rats were intraperitoneally injected with 90 mg/kg ketamine and 10 mg/kg thialazine while in a supine position. Under an operating microscope, three IVDs (Co6/7, Co8/9 and Co10/11) were punctured from the dorsal side with a 20 needle. The needle was passed through the center of the disc to the other side, rotated through 180°, and held in place for 10 s. Co7/8 and Co9/10 were used as the intact self‐control disc, as confirmed by magnetic resonance imaging [MRI] during IDD modeling. After the operation, the wound was covered with gauze and standard postoperative procedures were performed. Two weeks later, IDD was determined by MRI and Pfirrmann grading (Cheng et al., 2018). After successful modeling, 100 μl of lentivirus (Hanbio, Shanghai, China) was extracted with a Hamilton microinjector and injected into the NPC mass of the IVD. The control animals were injected with the same volume of normal saline, and the IDD of all animals was monitored weekly. The rats were used as NC, subjected to IDD modeling, or subjected to IDD and then transfected with oe‐NC, oe‐p300, oe‐NC + DMSO, oe‐p300 + Wnt/β‐catenin agonist 1, oe‐p300 + DMSO, or oe‐p300 + CQ treatment. The overexpression vectors were lentiviruses (Invitrogen). DMSO (Sigma) was used to dissolve Wnt/β‐catenin agonist 1 and CQ and was used as vehicle treatment in the NC group. Wnt/β‐catenin agonist 1 (MCE, Cat. No.: HY‐114321) and the autophagy inhibitor chloroquine (CQ; Sigma‐C6628) were injected into the rats after obtained overexpression. The lentiviral transduction complex was diluted according to the instructions of the En‐transterTM‐in vivo reagent. After the IDD rats were anesthetized, we made a small incision to expose the previously punctured IVDs. Next, 2 μl volumes containing Wnt/β‐catenin agonist 1, chloroquine, or oe‐p300 lentivirus‐transduced cells were slowly injected into the punctured disc. The injections were repeated 4 weeks later. 8 weeks after the injury, all rats were euthanized by carbon dioxide inhalation. The IVD tissues were collected, with the surrounding excess muscle and fat removed, and stored in liquid nitrogen until the subsequent Western blot assay and RT‐qPCR.

### 4.15 Safranin O‐fast green staining

Safranin O‐fast green staining was applied to examine cartilage tissue and collagen fibers. Paraffin sections were dewaxed and immersed in 75% alcohol. Next, the sections were stained with safranin dye solution for 1–2 h, and the sections were then decolorized in gradient alcohol. The sections were stained with fast green staining solution and dehydrated in an ethanol series to anhydrous ethanol. After permeabilizing with xylene for 5 min, the sections were sealed with neutral gum, followed by microscopic examination, and image acquisition and analysis.

### 4.16 TUNEL staining

TUNEL staining was used to measure the cell apoptosis in NP tissues. Paraffin sections were routinely dewaxed and rehydrated, washed with distilled water, soaked in 0.1 mM citric acid buffer, and the washed with water. The sections were rapidly cooled down by addition of 0.1 M Tris–HCl (containing 3% BSA and 20% calf serum). The TUNEL reaction mixture was dripped onto the sections, and the samples were incubated in a wet box at 37°C for 1 h. The apoptotic cells stained with yellow‐green fluorescence (FITC) were observed under fluorescence microscopy. Four visual fields were randomly selected from each sample for examination and photographing under high‐power microscopy.

### 4.17 Statistical analysis

All data were processed by SPSS 21.0 statistical software (SPSS, IBM). The measurement data, obtained from three independent experiments, were expressed as mean ± standard deviation. Comparison between two groups was conducted using independent sample *t*‐test, and comparison between multiple groups by one‐way analysis of variance (ANOVA), followed by Tukey post hoc test. Pearson's correlation analysis was used to analyze the correlations between indicators. The difference was statistically significant at *p* < 0.05.

## AUTHOR CONTRIBUTIONS

Yingjie Hao, Zhinan Ren, Lei Yu, and Guangduo Zhu designed study and collated data. Yingjie Hao, Panke Zhang, and Jian Zhu carried out data analyses and produced initial draft of manuscript. Zhinan Ren and Shuyan Cao contributed to drafting manuscript. All authors read and approved final manuscript.

## CONFLICT OF INTEREST

The authors declare that they have no competing interests.

## Supporting information


**Figure S1**.

Table S1

Table S2

Table S3

Table S4

## Data Availability

All data generated or analyzed during the current study are included in this published article.
