# Supplementary material for: p300 arrests intervertebral disc degeneration by regulating the FOXO3/Sirt1/Wnt/β‐catenin axis
Source: Aging Cell. 2022 Jul 30;21(8):e13677. doi: 10.1111/acel.13677 (PMC9381896; doi:10.1111/acel.13677)
Supplement: Supplementary file 1 — Figure S1. [file ACEL-21-e13677-s004.docx]

**
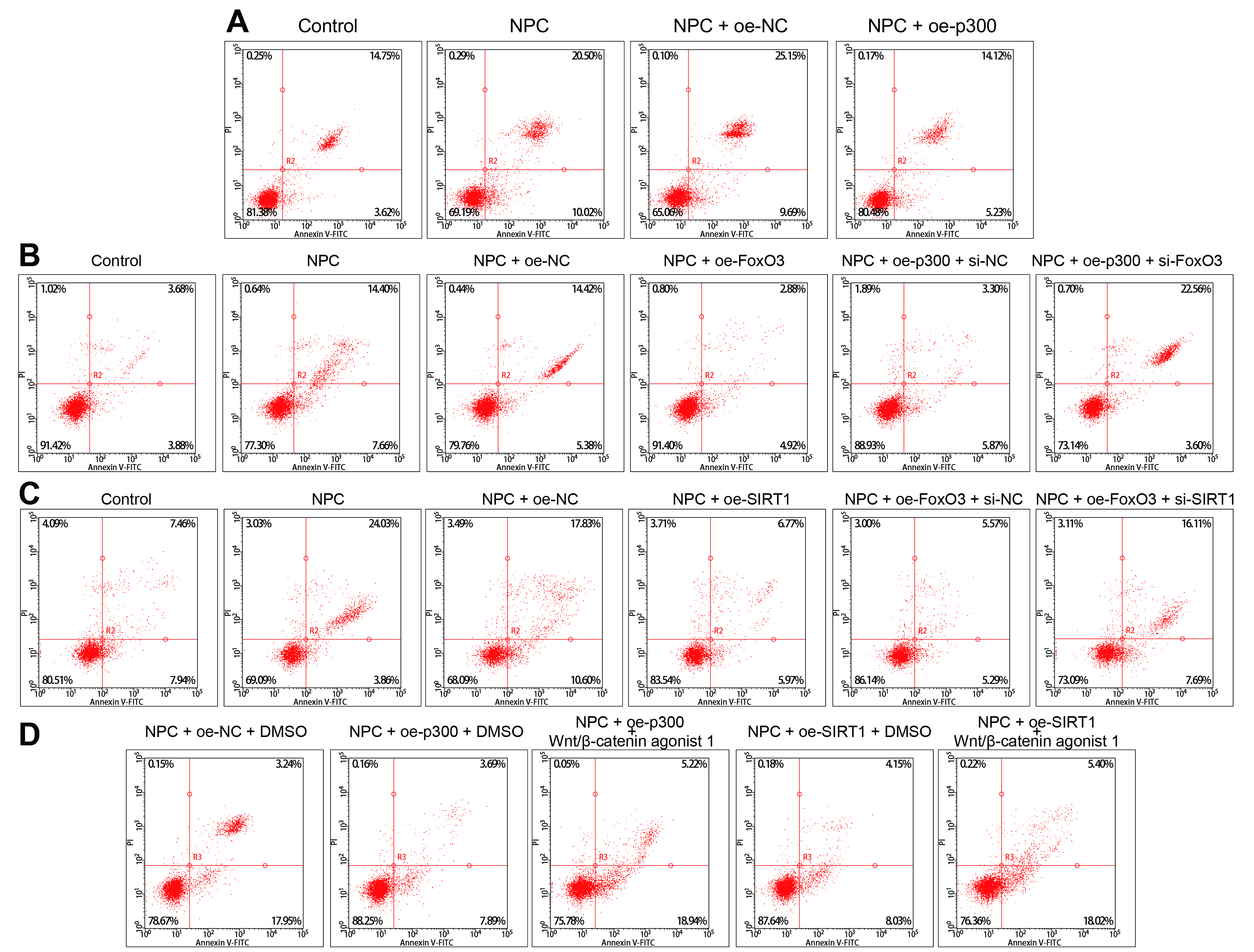
**

**Figure S1** The flow cytometric images of apoptosis of NPCs.

A, The apoptosis of NPCs in response to oe-p300. B, The apoptosis of NPCs in response to oe-FOXO3, oe-p300, or oe-p300 + si-FOXO3. C, The apoptosis of NPCs in response to oe-Sirt1, oe-FOXO3, or oe-FOXO3 + si-Sirt1. D, The apoptosis of NPCs in response to oe-p300 + Wnt/β-catenin agonist 1, oe-Sirt1, or oe-Sirt1 + Wnt/β-catenin agonist 1.

**
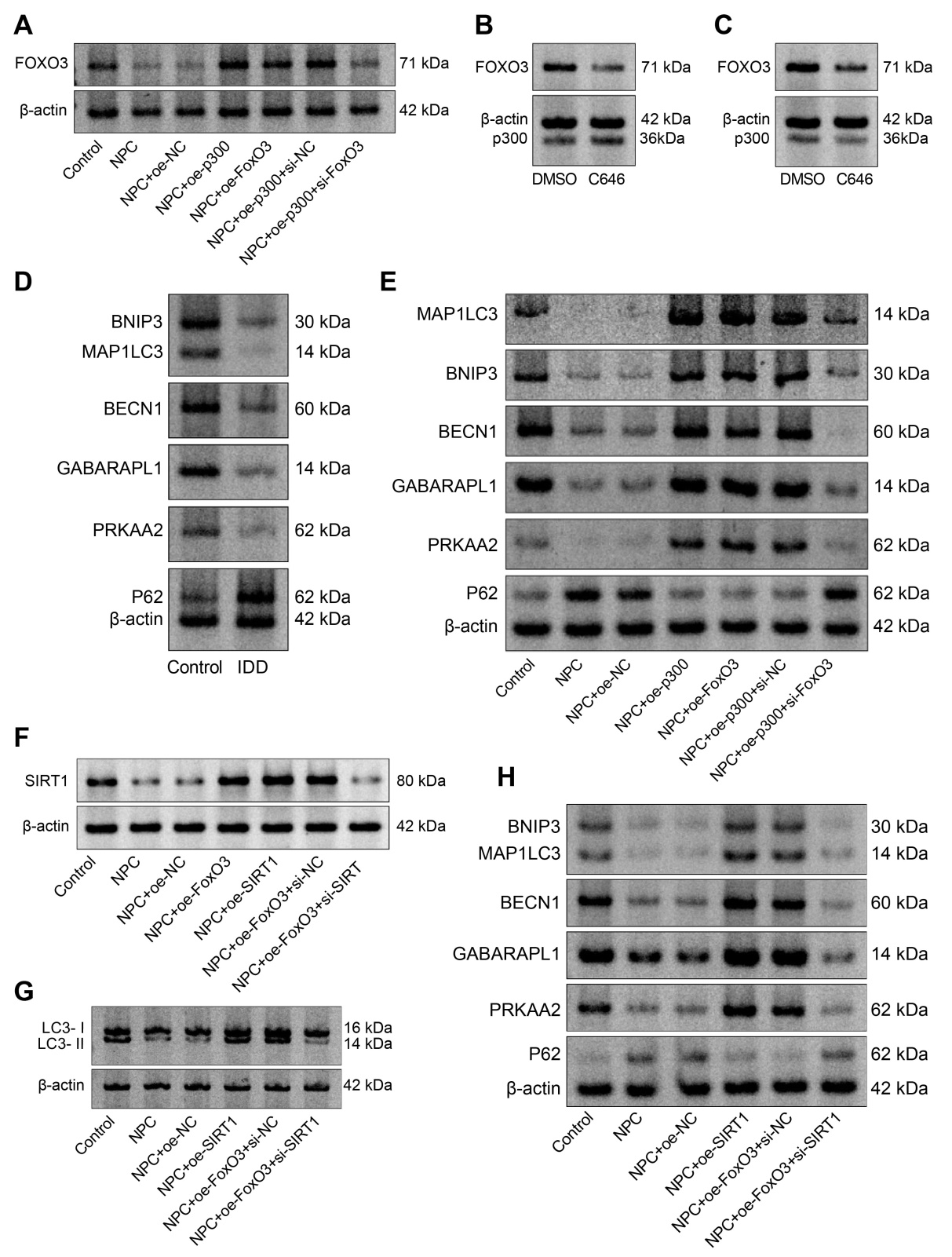
**

**Figure S2** Representative protein bands of Figure 2, 3, 4, and 5 (A, Figure 2D. B, Figure 2G. C, Figure 2H. D, Figure 3B. E, Figure 3E. F, Figure 4I. G, Figure 5C. H, Figure 5D).

**
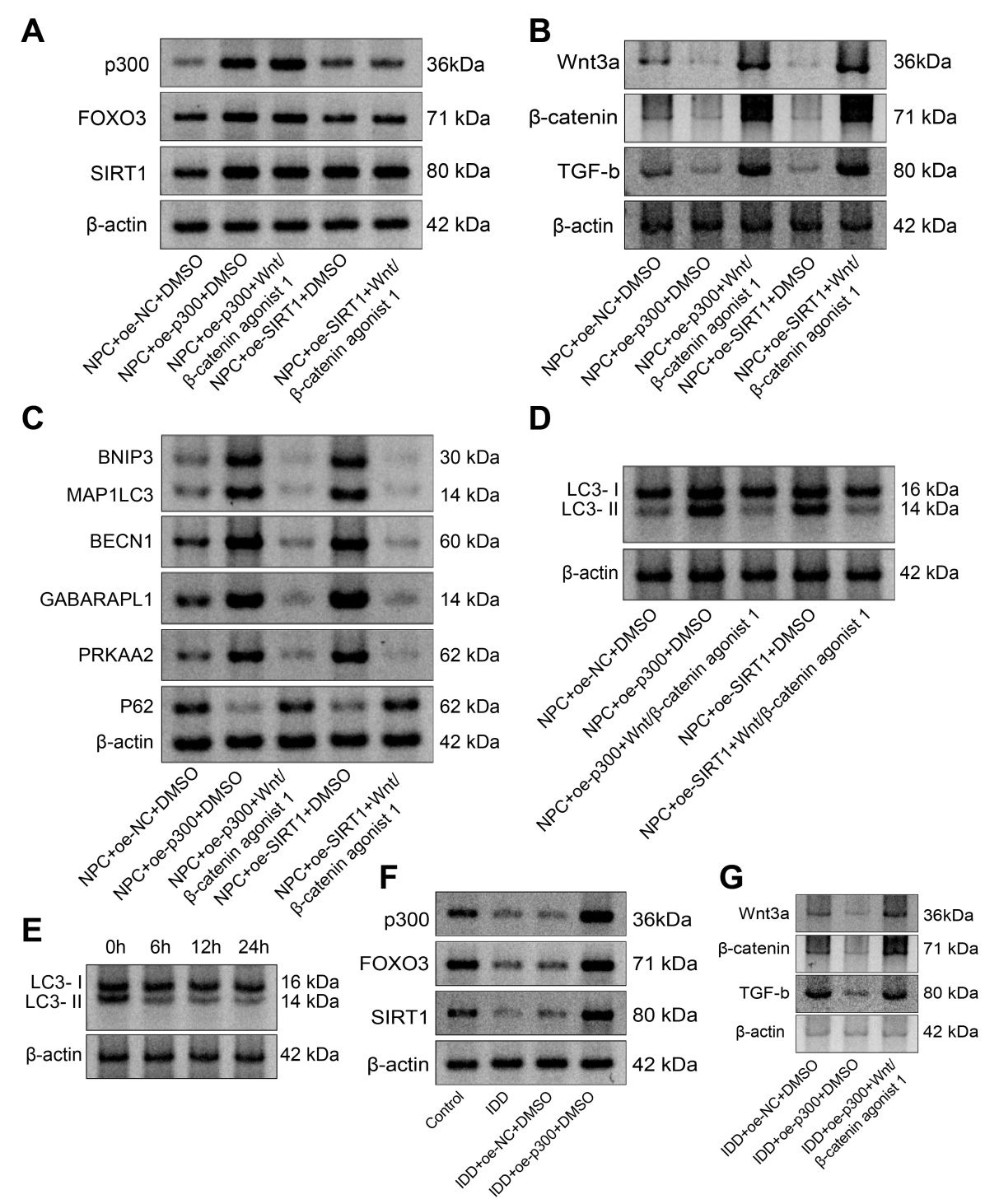
**

**Figure S3** Representative protein bands of Figure 6, and 7 (A, Figure 6A. B, Figure 6B. C, Figure 6G. D, Figure 6H. E, Figure 7A. F, Figure 7B. G, Figure 7C).

**
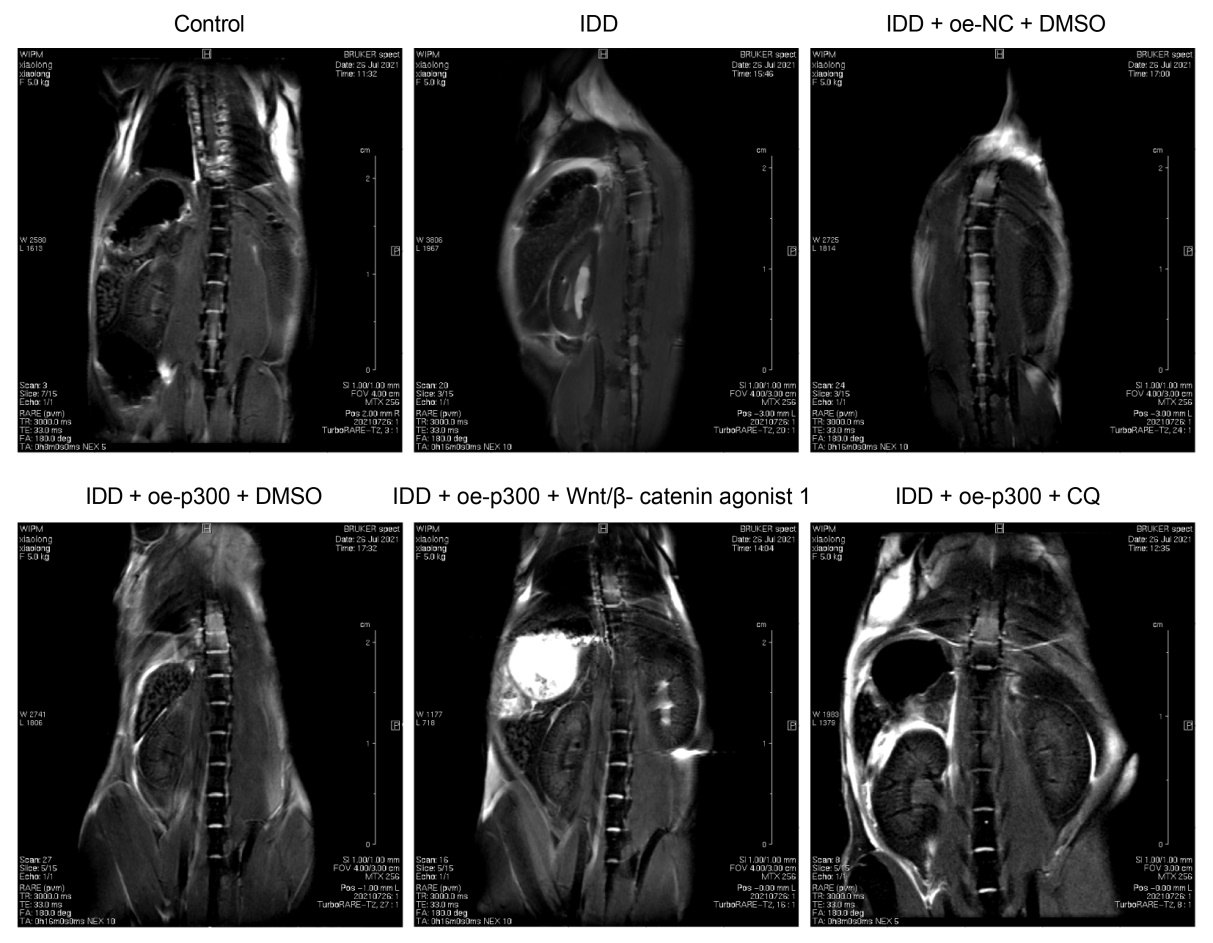
**

**Figure S4** IDD in rats determined by the MRI method with Pfirrmann grading.

**
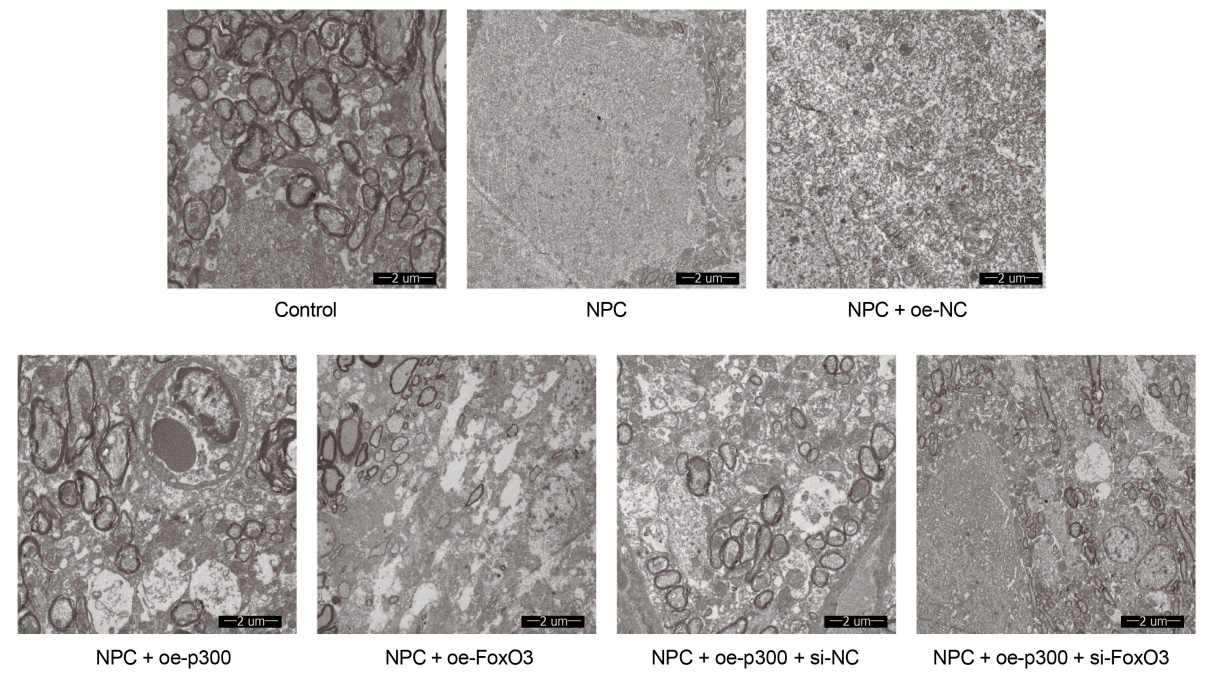
**

**Figure S5** Autophagosomes in NPCs of rat IVD tissues as observed by TEM.

**
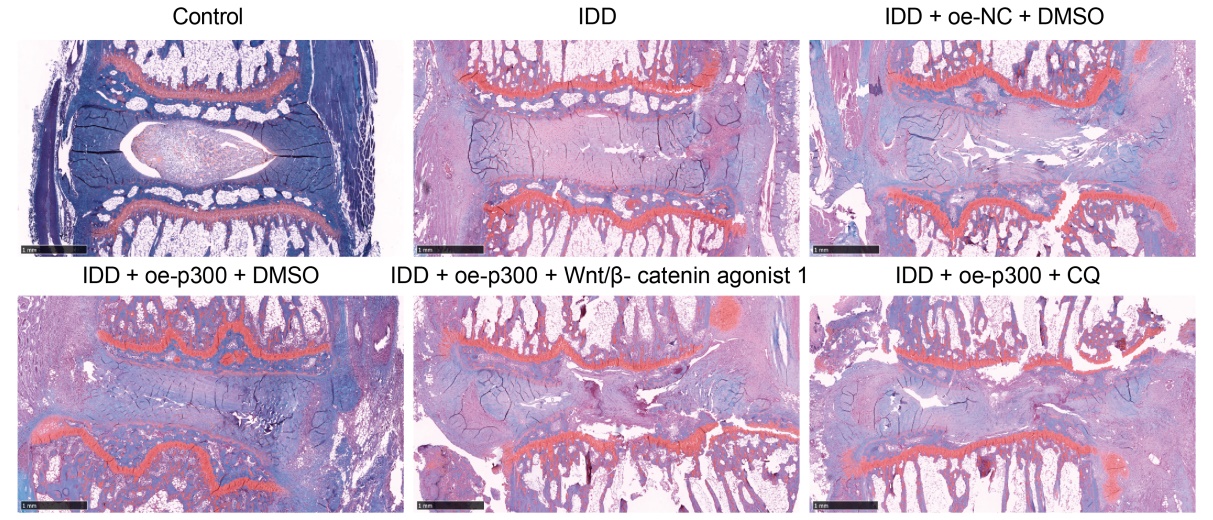
**

**Figure S6** Safranin O-fast green staining for the pathological changes of IVD tissues.
