# Supplementary material for: p300 arrests intervertebral disc degeneration by regulating the FOXO3/Sirt1/Wnt/β‐catenin axis
Source: Aging Cell. 2022 Jul 30;21(8):e13677. doi: 10.1111/acel.13677 (PMC9381896; doi:10.1111/acel.13677)
Supplement: Supplementary file 2 — Table S1 [file ACEL-21-e13677-s001.docx]

**Table S1** MRI grading criteria of Pfirrmann for the degree of IVDD

| Specimen | Grading | Signal changes of intervertebral disc structure | Annulus fibrosus and nucleus pulposus boundary | Changes in disc height |
| --- | --- | --- | --- | --- |
| Control (58) | I (36 cases) | Uniform white high signal | Clear | Normal |
|  | II (22 cases) | Uneven white signal | Clear | Normal |
| Experimental (58) | III (13 cases) | Uneven gray white signal | Unclear | Normal/slightly reduced |
|  | IV (20 cases) | Uneven black low signal | Disappear | Moderately reduced |
|  | V (25 cases) | Uneven black low signal | Disappear | intervertebral space collapse |

Note: MRI, magnetic resonance imaging; IVDD, intervertebral disc degeneration.
