# Supplementary material for: p300 arrests intervertebral disc degeneration by regulating the FOXO3/Sirt1/Wnt/β‐catenin axis
Source: Aging Cell. 2022 Jul 30;21(8):e13677. doi: 10.1111/acel.13677 (PMC9381896; doi:10.1111/acel.13677)
Supplement: Supplementary file 3 — Table S2 [file ACEL-21-e13677-s005.docx]

**Table S2** Clinical information of IVDD patients and healthy controls

| Number | Group | Sex | Age | Pathology | PfⅠrrmann |
| --- | --- | --- | --- | --- | --- |
| 1 | Control | male | 60 | thoracolumbar fractures | Ⅰ |
| 2 | Control | female | 46 | thoracolumbar fractures | Ⅱ |
| 3 | Control | male | 41 | thoracolumbar fractures | Ⅰ |
| 4 | Control | female | 60 | thoracolumbar fractures | Ⅰ |
| 5 | Control | male | 52 | thoracolumbar fractures | Ⅰ |
| 6 | Control | female | 50 | thoracolumbar fractures | Ⅰ |
| 7 | Control | male | 48 | thoracolumbar fractures | Ⅰ |
| 8 | Control | male | 62 | thoracolumbar fractures | Ⅱ |
| 9 | Control | male | 46 | thoracolumbar fractures | Ⅰ |
| 10 | Control | female | 43 | thoracolumbar fractures | Ⅱ |
| 11 | Control | male | 49 | thoracolumbar fractures | Ⅱ |
| 12 | Control | female | 55 | thoracolumbar fractures | Ⅰ |
| 13 | Control | male | 43 | thoracolumbar fractures | Ⅰ |
| 14 | Control | female | 50 | thoracolumbar fractures | Ⅰ |
| 15 | Control | male | 52 | thoracolumbar fractures | Ⅰ |
| 16 | Control | female | 55 | thoracolumbar fractures | Ⅱ |
| 17 | Control | female | 39 | thoracolumbar fractures | Ⅱ |
| 18 | Control | male | 53 | thoracolumbar fractures | Ⅱ |
| 19 | Control | female | 50 | thoracolumbar fractures | Ⅰ |
| 20 | Control | male | 50 | thoracolumbar fractures | Ⅱ |
| 21 | Control | female | 58 | thoracolumbar fractures | Ⅱ |
| 22 | Control | male | 57 | thoracolumbar fractures | Ⅰ |
| 23 | Control | female | 61 | thoracolumbar fractures | Ⅰ |
| 24 | Control | male | 37 | thoracolumbar fractures | Ⅰ |
| 25 | Control | female | 56 | thoracolumbar fractures | Ⅰ |
| 26 | Control | female | 55 | thoracolumbar fractures | Ⅱ |
| 27 | Control | male | 51 | thoracolumbar fractures | Ⅱ |
| 28 | Control | female | 48 | thoracolumbar fractures | Ⅱ |
| 29 | Control | male | 52 | thoracolumbar fractures | Ⅰ |
| 30 | Control | female | 46 | thoracolumbar fractures | Ⅱ |
| 31 | Control | female | 50 | scoliosis | Ⅱ |
| 32 | Control | female | 61 | scoliosis | Ⅰ |
| 33 | Control | male | 49 | scoliosis | Ⅰ |
| 34 | Control | female | 61 | scoliosis | Ⅰ |
| 35 | Control | male | 60 | scoliosis | Ⅰ |
| 36 | Control | female | 35 | scoliosis | Ⅱ |
| 37 | Control | male | 46 | scoliosis | Ⅱ |
| 38 | Control | female | 43 | scoliosis | Ⅰ |
| 39 | Control | male | 46 | scoliosis | Ⅱ |
| 40 | Control | female | 54 | scoliosis | Ⅱ |
| 41 | Control | female | 56 | scoliosis | Ⅰ |
| 42 | Control | male | 44 | scoliosis | Ⅰ |
| 43 | Control | female | 53 | scoliosis | Ⅰ |
| 44 | Control | male | 46 | scoliosis | Ⅰ |
| 45 | Control | male | 42 | scoliosis | Ⅱ |
| 46 | Control | male | 59 | scoliosis | Ⅱ |
| 47 | Control | female | 64 | scoliosis | Ⅱ |
| 48 | Control | male | 66 | scoliosis | Ⅱ |
| 49 | Control | female | 51 | scoliosis | Ⅰ |
| 50 | Control | male | 50 | scoliosis | Ⅱ |
| 51 | Control | male | 53 | scoliosis | Ⅱ |
| 52 | Control | male | 53 | scoliosis | Ⅱ |
| 53 | Control | male | 49 | scoliosis | Ⅰ |
| 54 | Control | female | 56 | scoliosis | Ⅱ |
| 55 | Control | male | 40 | scoliosis | Ⅱ |
| 56 | Control | female | 46 | scoliosis | Ⅰ |
| 57 | Control | male | 69 | scoliosis | Ⅰ |
| 58 | Control | male | 55 | scoliosis | Ⅰ |
| 1 | IVDD | male | 52 | lumbar disc herniation | Ⅴ |
| 2 | IVDD | male | 35 | lumbar disc herniation | Ⅴ |
| 3 | IVDD | male | 46 | lumbar disc herniation | Ⅴ |
| 4 | IVDD | male | 64 | lumbar disc herniation | Ⅴ |
| 5 | IVDD | female | 54 | lumbar disc herniation | Ⅴ |
| 6 | IVDD | female | 50 | lumbar disc herniation | Ⅴ |
| 7 | IVDD | male | 64 | lumbar disc herniation | Ⅲ |
| 8 | IVDD | female | 66 | lumbar disc herniation | Ⅳ |
| 9 | IVDD | male | 62 | lumbar disc herniation | Ⅳ |
| 10 | IVDD | male | 49 | lumbar disc herniation | Ⅳ |
| 11 | IVDD | male | 52 | lumbar disc herniation | Ⅴ |
| 12 | IVDD | female | 55 | lumbar disc herniation | Ⅳ |
| 13 | IVDD | male | 61 | lumbar disc herniation | Ⅲ |
| 14 | IVDD | male | 49 | lumbar disc herniation | Ⅲ |
| 15 | IVDD | male | 46 | lumbar disc herniation | Ⅲ |
| 16 | IVDD | female | 51 | lumbar disc herniation | Ⅳ |
| 17 | IVDD | male | 54 | lumbar disc herniation | Ⅳ |
| 18 | IVDD | female | 54 | lumbar disc herniation | Ⅲ |
| 19 | IVDD | male | 45 | lumbar disc herniation | Ⅴ |
| 20 | IVDD | female | 43 | lumbar disc herniation | Ⅴ |
| 21 | IVDD | female | 51 | lumbar disc herniation | Ⅴ |
| 22 | IVDD | female | 60 | lumbar disc herniation | Ⅳ |
| 23 | IVDD | male | 55 | lumbar disc herniation | Ⅳ |
| 24 | IVDD | male | 59 | lumbar disc herniation | Ⅲ |
| 25 | IVDD | female | 56 | lumbar disc herniation | Ⅳ |
| 26 | IVDD | male | 56 | lumbar disc herniation | Ⅳ |
| 27 | IVDD | male | 50 | lumbar disc herniation | Ⅳ |
| 28 | IVDD | female | 42 | lumbar disc herniation | Ⅴ |
| 29 | IVDD | male | 54 | lumbar disc herniation | Ⅳ |
| 30 | IVDD | male | 63 | lumbar disc herniation | Ⅳ |
| 31 | IVDD | female | 48 | lumbar disc herniation | Ⅴ |
| 32 | IVDD | female | 43 | lumbar disc herniation | Ⅴ |
| 33 | IVDD | female | 53 | lumbar disc herniation | Ⅴ |
| 34 | IVDD | male | 41 | lumbar disc herniation | Ⅲ |
| 35 | IVDD | female | 46 | lumbar disc herniation | Ⅳ |
| 36 | IVDD | female | 56 | lumbar disc herniation | Ⅲ |
| 37 | IVDD | male | 61 | lumbar disc herniation | Ⅳ |
| 38 | IVDD | female | 55 | lumbar disc herniation | Ⅴ |
| 39 | IVDD | male | 43 | lumbar disc herniation | Ⅲ |
| 40 | IVDD | male | 42 | lumbar disc herniation | Ⅲ |
| 41 | IVDD | female | 54 | lumbar disc herniation | Ⅴ |
| 42 | IVDD | male | 58 | lumbar disc herniation | Ⅲ |
| 43 | IVDD | female | 56 | lumbar disc herniation | Ⅳ |
| 44 | IVDD | male | 48 | lumbar disc herniation | Ⅳ |
| 45 | IVDD | male | 44 | lumbar disc herniation | Ⅴ |
| 46 | IVDD | female | 47 | lumbar disc herniation | Ⅳ |
| 47 | IVDD | male | 55 | lumbar disc herniation | Ⅴ |
| 48 | IVDD | male | 65 | lumbar disc herniation | Ⅴ |
| 49 | IVDD | female | 54 | lumbar disc herniation | Ⅴ |
| 50 | IVDD | male | 53 | lumbar disc herniation | Ⅴ |
| 51 | IVDD | female | 56 | lumbar disc herniation | Ⅳ |
| 52 | IVDD | male | 50 | lumbar disc herniation | Ⅳ |
| 53 | IVDD | male | 53 | lumbar disc herniation | Ⅲ |
| 54 | IVDD | male | 44 | lumbar disc herniation | Ⅴ |
| 55 | IVDD | female | 35 | lumbar disc herniation | Ⅴ |
| 56 | IVDD | male | 60 | lumbar disc herniation | Ⅴ |
| 57 | IVDD | male | 57 | lumbar disc herniation | Ⅴ |
| 58 | IVDD | female | 56 | lumbar disc herniation | Ⅲ |
