# Supplementary material for: p300 arrests intervertebral disc degeneration by regulating the FOXO3/Sirt1/Wnt/β‐catenin axis
Source: Aging Cell. 2022 Jul 30;21(8):e13677. doi: 10.1111/acel.13677 (PMC9381896; doi:10.1111/acel.13677)
Supplement: Supplementary file 4 — Table S3 [file ACEL-21-e13677-s003.docx]

**Table S3** Primer sequences for RT-qPCR

| Gene | Sequences |
| --- | --- |
| p300 (*Homo*) (BC053889.1) | F: 5-TTGTGAAGAGCCCCATGGAT-3 |
|  | R: 5-GCTTTGCATCACTGGGTCAA-3 |
| p300 (*Rat*) (AB066220.1) | F: 5-AGACAGGCTTGACTTCTCCC-3 |
|  | R: 5-TTGGGTACTGCATGTTTGGC-3 |
| FOXO3 (*Homo*) (NM001455.4) | F: 5-TCCGCGATCCTGTACGTGG-3 |
|  | R: 5-ACAGAGTGAGCCGTTTGTCC-3 |
| FOXO3 (*Rat*) (NM001106395.1) | F: 5-TCCTGGCGGGCTTATGCAG-3 |
|  | R: 5-GACATCATTGGGTCGTTGCG-3 |
| Sirt1 (*Homo*) (NM012238.5) | F: 5-TGCCGGAAACAATACCTCCA-3 |
|  | R: 5-AGACACCCCAGCTCCAGTTA-3 |
| SIRT1 (Rat) (XM_039098755.1) | F: 5-TACCAGAAACAATTCCTCCA-3 |
|  | R: 5-AGAAACCCCAGCTCCAGTCA-3 |
| GAPDH (*Homo*) | F: 5-AGCCACATCGCTCAGACAC-3 |
|  | R: 5-GCCCAATACGACCAAATCC-3 |
| GAPDH (*Rat*) (NM017008.4) | F: 5-TGTGAACGGATTTGGCCGTA-3 |
|  | R: 5-GATGGTGATGGGTTTCCCGT-3 |

Note: FOXO3, Forkhead box O3; Sirt1, sirtuin 1; GAPDH, glyceraldehyde-3-phosphate dehydrogenase; RT-qPCR, reverse transcription-quantitative polymerase chain reaction
