# Supplementary material for: p300 arrests intervertebral disc degeneration by regulating the FOXO3/Sirt1/Wnt/β‐catenin axis
Source: Aging Cell. 2022 Jul 30;21(8):e13677. doi: 10.1111/acel.13677 (PMC9381896; doi:10.1111/acel.13677)
Supplement: Supplementary file 5 — Table S4 [file ACEL-21-e13677-s002.docx]

**Table S4** PCR primers for SIRT1 promoter

| Primer name | Sequences |
| --- | --- |
| SIRT1-PRO-F1 | GTCCTAATGCTCTCCCTCCC |
| SIRT1-PRO-R1 | GGTTCTGGCCATACTGCATC |
| SIRT1-PRO-F2 | AGGAGGGAATTCACACACGT |
| SIRT1-PRO-R2 | ACTCTCCTCACACCGTTCTG |

Note: Sirt1, sirtuin 1; PCR, polymerase chain reaction
